# Multiple Lytic Origins of Replication Are Required for Optimal Gammaherpesvirus Fitness In Vitro and In Vivo

**DOI:** 10.1371/journal.ppat.1005510

**Published:** 2016-03-23

**Authors:** Christine Sattler, Beatrix Steer, Heiko Adler

**Affiliations:** Research Unit Gene Vectors, Helmholtz Zentrum München - German Research Center for Environmental Health (GmbH), Munich, Germany; Baylor College of Medicine, UNITED STATES

## Abstract

An unresolved question in herpesvirus biology is why some herpesviruses contain more than one lytic origin of replication (oriLyt). Using murine gammaherpesvirus 68 (MHV-68) as model virus containing two oriLyts, we demonstrate that loss of either of the two oriLyts was well tolerated in some situations but not in others both in vitro and in vivo. This was related to the cell type, the organ or the route of inoculation. Depending on the cell type, different cellular proteins, for example Hexim1 and Rbbp4, were found to be associated with oriLyt DNA. Overexpression or downregulation of these proteins differentially affected the growth of mutants lacking either the left or the right oriLyt. Thus, multiple oriLyts are required to ensure optimal fitness in different cell types and tissues.

## Author Summary

Herpesviruses show two stages in their life cycle: lytic replication and latency. Lytic DNA replication is initiated at a defined site on the viral genome, the so-called lytic origin of replication (oriLyt). While some herpesviruses have a single oriLyt, others have multiple oriLyts. Why some herpesviruses need more than one oriLyt is not known. This study demonstrates that the presence of multiple oriLyts enables gammaherpesviruses to efficiently establish infection in different cell or tissue types and during different phases of the viral life cycle. Depending on the cell type, different cellular proteins were found to be associated with oriLyt DNA, and overexpression or downregulation of these proteins differentially affected the growth of viruses containing only a single oriLyt. Thus, multiple oriLyts ensure optimal viral fitness in different cell types and tissues.

## Introduction

Herpesviruses show two stages in their life cycle: lytic replication and latency. Lytic DNA replication is initiated at a defined site on the viral genome, the lytic origin of replication (oriLyt). While some herpesviruses, for example human cytomegalovirus (HCMV), have a single oriLyt, others have multiple oriLyts [1]. Why some herpesviruses need more than one oriLyt is not known [2].

Across different herpesvirus family members, oriLyts may vary in size and in complexity, but are usually characterized by the presence of binding sites for transcription factors and repeat sequences [3]. "Trans"-acting factors, usually multi-protein complexes, are necessary for efficient oriLyt-dependent DNA replication. During the lytic cycle, a multi-protein complex is formed at the oriLyt and initiates the replication process [4]. This complex is composed of viral proteins, which first form a pre-replication complex that is then recruited to the oriLyt, binds to it and subsequently becomes the replication initiation complex. The viral proteins are conserved among the various herpesviruses, and are often referred to as the six core replication proteins: DNA polymerase, processivity factor, helicase, primase, primase-associated factor and ssDNA binding protein. In addition to these factors, each herpesvirus needs at least one origin-binding protein, e.g. Zta for Epstein-Barr virus (EBV) and Rta and bZIP for Kaposi's sarcoma-associated herpesvirus (KSHV) [5]. Besides viral proteins, cellular proteins are also involved in the complex. While the identity of viral proteins is relatively well established, not all of the cellular proteins are known [6]. For KSHV, Topoisomerase I and II, for example, have been shown to interact with the oriLyt [6]. Using depletion of Topoisomerase I and II by shRNA-mediated "gene silencing" or by chemical inhibition, lytic replication of KSHV could be significantly inhibited [7].

The two known human gammaherpesviruses (γHV) EBV and KSHV belong to those herpesviruses that have more than one oriLyt, namely two [8,9]. The prototypic γ1-herpesvirus EBV is associated with lymphomas and nasopharyngeal carcinoma [10]. KSHV, a γ2-herpesvirus, is associated with lymphoproliferative disorders and Kaposi’s sarcoma [11]. In Kaposi's sarcoma lesions, most of the endothelial-derived spindle cells are latently infected with KSHV. In some cells, however, there is also spontaneous lytic replication which might contribute to viral spread and thereby to the preservation of the pool of latently infected cells [12]. In addition, soluble factors are produced during lytic replication which promote tumorigenesis by paracrine mechanisms [13,14]. Consistent with these findings is the observation that treatment with ganciclovir which inhibits lytic replication limited the development of Kaposi's sarcoma [15]. It was therefore postulated that for KSHV, lytic replication and continuous re-infection of naive cells are of great importance for tumorigenesis [6]. To gain insight into why γHV like KSHV need two oriLyts may thus not only lead to a better understanding of oriLyt-dependent lytic replication in general but might also aid in the development of new avenues for interference with herpesvirus lytic replication and disease development.

Although there are suitable cell culture systems to study EBV and KSHV lytic replication, they are rather inefficient when compared to other viruses. Murine gammaherpesvirus 68 (MHV-68) is also a member of the γHV and closely related to KSHV and EBV [16]. MHV-68 replicates well in tissue culture and infection of mice serves as a small animal model to investigate γHV pathogenesis [17]. Thus, it is a good model to study oriLyt-dependent lytic replication in vitro and in vivo. Importantly, since MHV-68 also contains two oriLyts [4,18,19], it represents a suitable model to approach the question why γHV need more than one oriLyt.

In the present study, we investigate the role of the oriLyts in the context of a γHV infection using MHV-68 mutants lacking either a functional left or right oriLyt. We find that in vitro, the efficiency of replication of the oriLyt mutants is dependent on the cell type. In vivo, we observe differences amongst the mutants with regard to acute lytic replication, latent viral load and reactivation capacity. Identification of oriLyt-bound cellular proteins reveals that, depending on the cell line, a different repertoire of proteins interacts with the respective oriLyt or the associated replication complex. Overexpression or downregulation of such proteins differentially affects the growth of mutants lacking either the left or the right oriLyt. Taken together, our data suggest that the presence of multiple oriLyts enables γHV to efficiently establish infection in different cell or tissue types and during different phases of the viral life cycle.

## Results

### Lytic growth of oriLyt mutants varies between cell types

**Fig 1:**
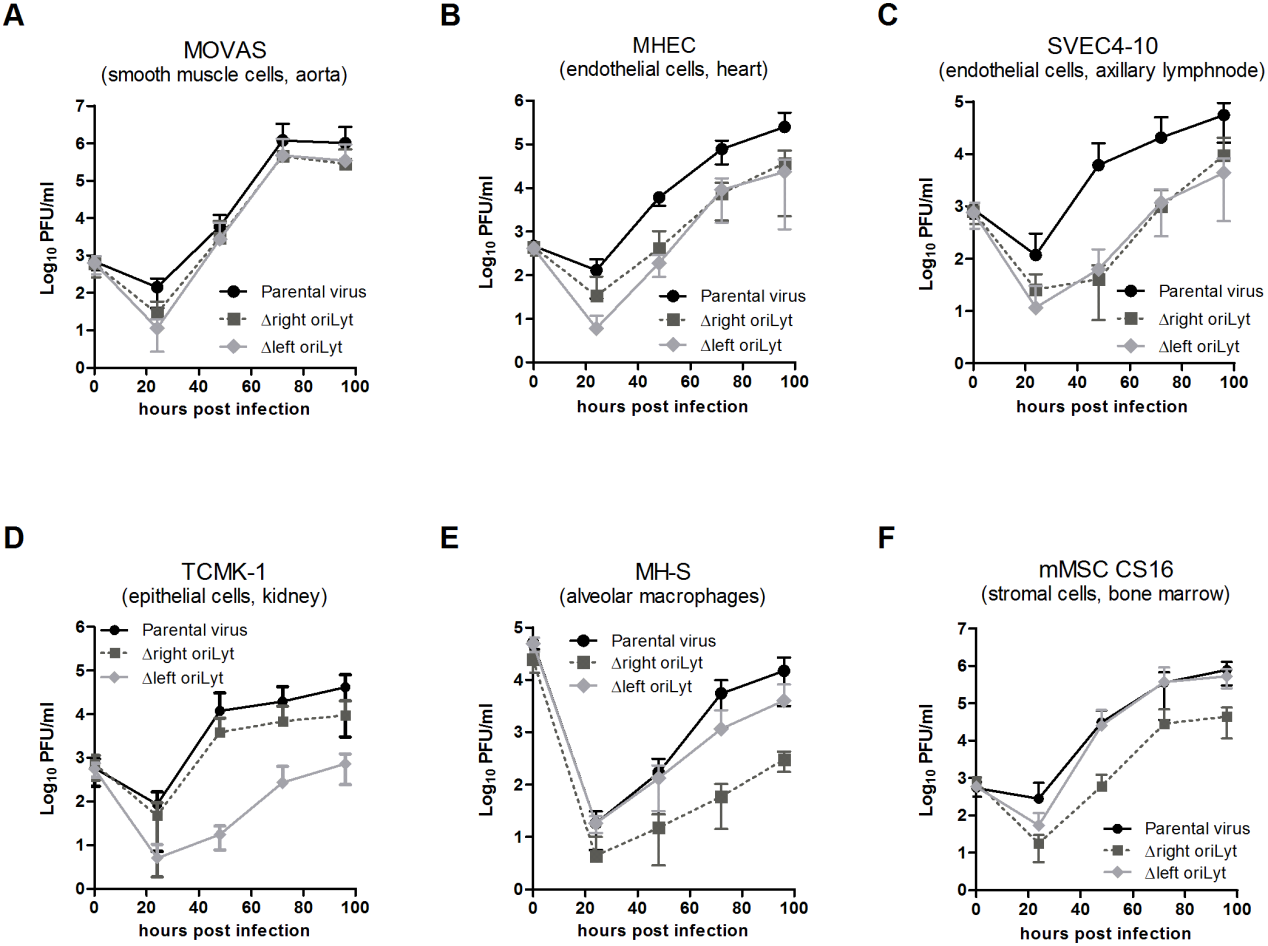
Virus replication in vitro. MOVAS (A), MHEC (B), SVEC 4–10 (C), TCMK-1 (D), MH-S (E) and CS16 stromal cells (F) were infected with the indicated viruses at an MOI of 0.01 (MOVAS, MHEC, SVEC 4–10, TCMK-1, CS16) or 1 (MH-S). Cells and cell culture supernatants were harvested at different time points p.i., and titers were determined by plaque assay on BHK-21 cells. Data shown are the means ± SD from three (SVEC 4–10 and MH-S), four (MOVAS and MHEC) or five (TCMK-1 and mMSC CS16) independent experiments.

Throughout the infection process in vivo, MHV-68 encounters various cell types in various tissues. This might pose specific challenges to the capacity of an oriLyt in these tissues and cells. In order to model this situation in vitro, we first analyzed the growth of mutants lacking the left or the right oriLyt (Fig A in S1 Text) in thirteen cell lines of different cell type and origin. In some cell lines, for example in the smooth muscle cell line MOVAS (Fig 1A), both oriLyt mutants attained similar titers as the parental virus. However, in other cell lines like MHEC and SVEC4-10 endothelial cells, both mutants showed a replication deficit compared to parental virus (Fig 1B and 1C). Interestingly, cell lines were also found in which only one but not the other mutant was impaired in lytic growth, indicating that the oriLyts are of varying importance in these cell lines. For example, the Δright oriLyt mutant attained titers comparable to parental virus in the epithelial cell line TCMK-1, whereas the Δleft oriLyt mutant showed a replication deficit of more than one order of magnitude in this cell line (Fig 1D). In contrast, in the alveolar macrophage cell line MH-S and in the mesenchymal stromal cell line CS16, only the Δright oriLyt mutant was impaired in lytic replication while the Δleft oriLyt mutant was not (Fig 1E and 1F). Table A in S1 Text summarizes the results for lytic growth of the mutants in comparison to parental virus in all tested cell lines. To demonstrate that the phenotypes observed with the oriLyt mutants were due to the deletion of the oriLyt and not to rearrangements outside of the mutated region or side effects of the deletion on neighbouring genes, ectopic revertants were constructed. In these revertants, the oriLyt-sequence has been re-inserted at an ectopic position while the original deletion is still present (Fig A in S1 Text). Thus, a complete reversion of the mutant phenotype clearly indicates that it was not due to disruption of other structures or to side effects on neighboring genes. We selected a cell line (MHEC) in which both oriLyt mutants displayed reduced growth, and tested the respective ectopic revertants. A shown in Fig B in S1 Text, both ectopic revertants showed a complete reversion of the phenotype of their respective mutant. Our results demonstrate that differences exist between the two oriLyt mutants regarding their capacity for lytic growth in specific cell lines.

### Reduced lung titers after infection with oriLyt mutants

**Fig 2:**
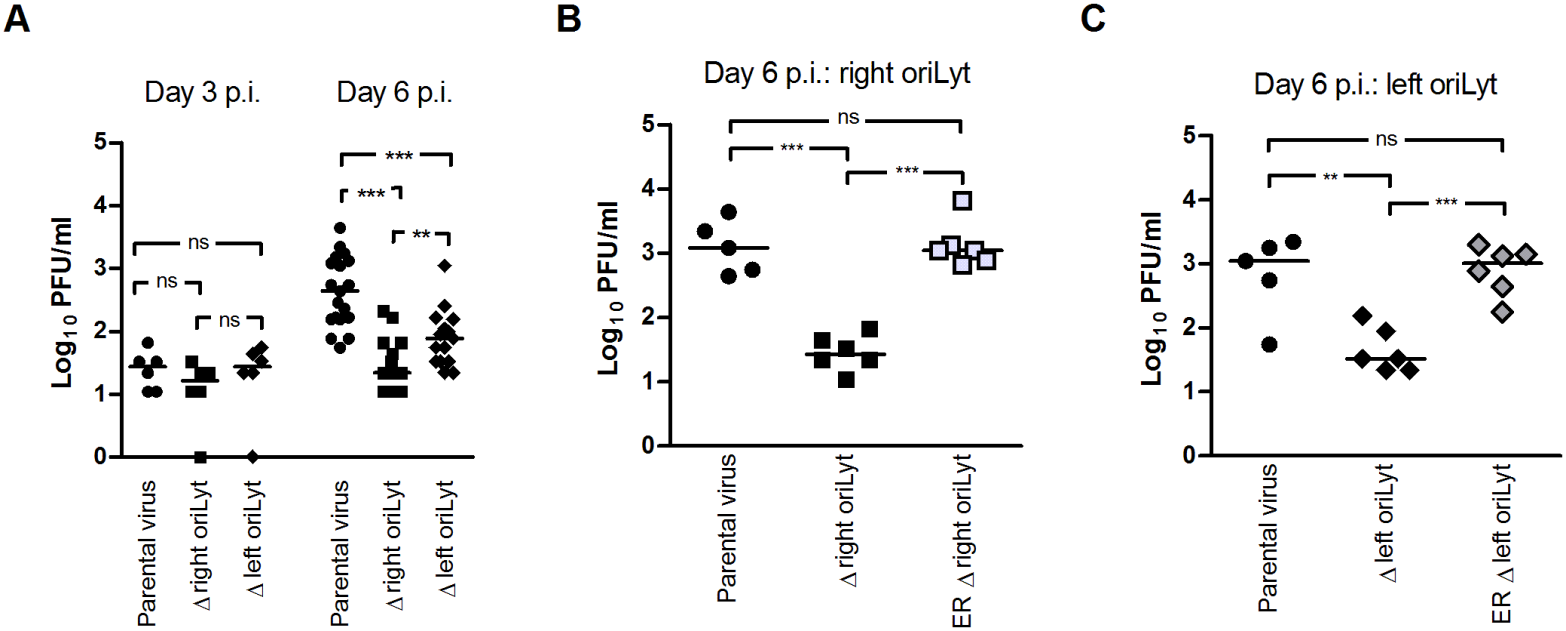
Lytic replication in the lungs after i.n. inoculation. C57BL/6 mice were inoculated i.n. with 1x 10^3^ PFU of the indicated viruses. Lungs of mice were harvested at day 3 after infection (parental virus or mutant viruses) and at day 6 after infection (parental or mutant viruses and ectopic revertants). Virus titers were determined from organ homogenates by plaque assay. Each symbol represents an individual mouse, and the bars represent the median value. (A) The data are compiled from two (day 3) and five (day 6 mutant viruses) or six (day 6 parental virus) independent experiments. (B and C) The data are compiled from two independent experiments in each case. The asterisks indicate a statistically significant difference between the groups (* P < 0.05; ** P < 0.01; *** P < 0.001).

To analyze whether the observed growth differences between the two oriLyt mutants are only an in vitro phenomenon, or whether the two oriLyts might have different functions also in vivo, C57BL/6 mice were inoculated intranasally (i.n.). Following i.n. inoculation, there is an acute phase of lytic virus replication which involves alveolar epithelial cells [17]. Virus titers were determined in lung homogenates by plaque assay at an early time point (day 3 p.i.) and at a time point when peak viral titers are usually reached (day 6 p.i.). No differences in viral titers were detected at day 3 after infection (Fig 2A). Consistent with results published previously by our group, viral titers in the lungs of mice infected with the Δright oriLyt mutant were significantly lower at day 6 p.i. when compared to parental virus (Fig 2A; [20]). Likewise, viral titers in mice infected with the Δleft oriLyt mutant were reduced; however, the titers were significantly higher than after infection with the Δright oriLyt mutant. Viral titers after infection with the ectopic revertants, both for the right oriLyt (Fig 2B) and for the left oriLyt (Fig 2C), were not significantly reduced when compared to parental virus. This indicated that the phenotypes observed with the oriLyt mutants were indeed due to the deletion of the oriLyt and not to rearrangements outside of the mutated region or side effects of the deletion on neighboring genes. Taken together, our results show that deletions of the right or the left oriLyt also differentially affect lytic replication in vivo.

### OriLyt deletions affect latency and reactivation

**Fig 3:**
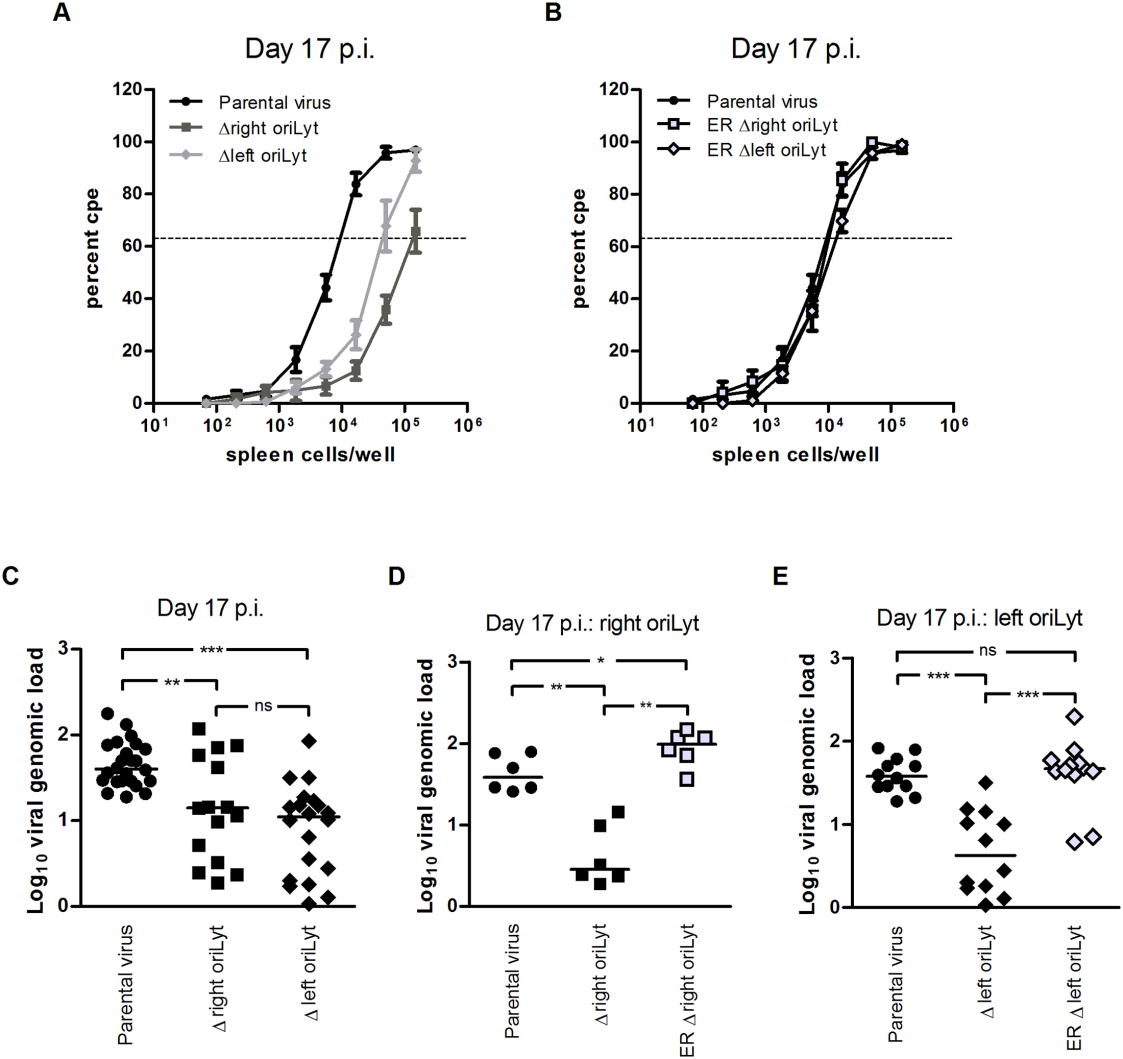
Latent infection in the spleen after i.n. inoculation. (A and B) ex vivo reactivation of splenocytes; (C–E) viral genomic load in the spleen. C57BL/6 mice were inoculated with 1 x 10^3^ PFU of the indicated viruses. At day 17 after infection, spleens were harvested. Single splenocyte suspensions were prepared and analyzed in the ex vivo reactivation assay or used for DNA isolation for real-time PCR analysis. Data shown in panel A are the means ± standard errors of the means pooled from six (parental virus) and five (mutant viruses) independent experiments. Data shown in panel B are pooled from two independent experiments. In each experiment, splenocytes from three mice per group were pooled. The dashed line indicates the point of 63.2% Poisson distribution, determined by nonlinear regression, which was used to calculate the frequency of cells reactivating lytic replication. The obtained values for reactivation frequency were statistically analyzed by Student’s t-test to calculate significances. Statistical significance was found between the group with parental virus and the group with the Δright oriLyt mutant (P = 0.0021), between the group with parental virus and the group with the Δleft oriLyt mutant (P = 0.004), and between the two groups with mutant virus (P = 0.0145). In panels C–E, each symbol represents an individual mouse, and the bars represent the median value. The data in panel C are compiled from six (parental virus) and five (mutant viruses) independent experiments. The data in panels D and E are from two independent experiments each. The asterisks in panels C–E indicate statistical significance between the groups (* P < 0.05; ** P < 0.01; *** P < 0.001).

To analyze the oriLyt mutants during the latent phase of infection, C57BL/6 mice were i.n. inoculated. After i.n. inoculation, the spleen is a major site of latently infected cells [17]. Thus, ex vivo reactivation of latently infected splenocytes and the viral genomic load in the spleen were determined 17 days after infection (early latency). At this stage, the majority of cells in the spleen harbouring MHV-68 are B cells [17]. Significantly fewer splenocytes reactivated from mice infected with the oriLyt mutants compared to those infected with parental virus, the number of reactivating splenocytes being lowest in mice infected with the Δright oriLyt mutant (Fig 3A). The frequency of reactivating splenocytes was 1 in 9849 for the parental virus. This number was significantly lower in mice infected with the Δright oriLyt mutant (1 in 250649; P = 0.0021 versus parental virus) and in mice infected with the Δleft oriLyt mutant (1 in 53122; P = 0.004 versus parental virus). In all experiments, the frequency of reactivating splenocytes from mice infected with the Δright oriLyt mutant was found to be significantly lower than in mice infected with the Δleft oriLyt mutant (P = 0.0145). Splenocytes from mice infected with the ectopic revertants showed a frequency of reactivation similar to splenocytes from mice infected with parental virus (Fig 3B). The viral genomic load in the spleens of infected mice was determined by real-time PCR. A significantly lower viral copy number was found in both groups infected with oriLyt mutant viruses when compared to mice infected with parental virus (Fig 3C). The copy numbers between the group infected with the Δright oriLyt mutant and the group infected with the Δleft oriLyt mutant were comparable. Thus, the reduced reactivation frequency of the Δleft oriLyt mutant might solely be due to the lower viral copy number while for the Δright oriLyt mutant, a defect in reactivation itself seems to be present additionally. Importantly, the viral copy numbers after infection with the ectopic revertants were not significantly reduced compared to the copy numbers after infection with parental virus, neither for the right oriLyt (Fig 3D) nor for the left oriLyt (Fig 3E). We also tested ex vivo reactivation and viral genomic load at day 42 post infection (late latency). Here, no reactivation could be detected and the viral genomic loads were comparable between the group with parental virus and the two groups with mutant viruses (8.3 ± 1.6, 10.1 ± 1.8 and 14.6 ± 4.4 copies gB/1000 copies L8 [means ± SEM; n = 6] for parental virus, Δright oriLyt mutant and Δleft oriLyt mutant, respectively). Our results regarding viral latency after i.n. inoculation demonstrate that both the deletion of the right or the left oriLyt affect latency establishment and the capacity to reactivate from early latency (day 17 post infection). Deletion of the right oriLyt had a stronger effect than deletion of the left oriLyt. The defect in latency establishment and reactivation of the oriLyt mutants might be the result of inefficient acute replication (Fig 2A) but could also be due to effects on latency establishment and reactivation itself. Although we cannot formally rule out the first option, we favor the second alternative for the following reasons: i) vaccination studies with MHV-68 suggest that efficient acute infection is not a mandatory step for latency establishment [21,22], and ii) the level of latently infected cells in the spleen is largely independent of the dose used for inoculation—in case of i.n. inoculation over a range from 4 x 10^1^ to 4 x 10^5^ PFU [23]. In our study, the titers of the oriLyt mutants during acute infection were within this range and were approx. 20-fold lower when compared to parental virus (Fig 2A).

### The need for two oriLyts is related to the inoculation route

**Fig 4:**
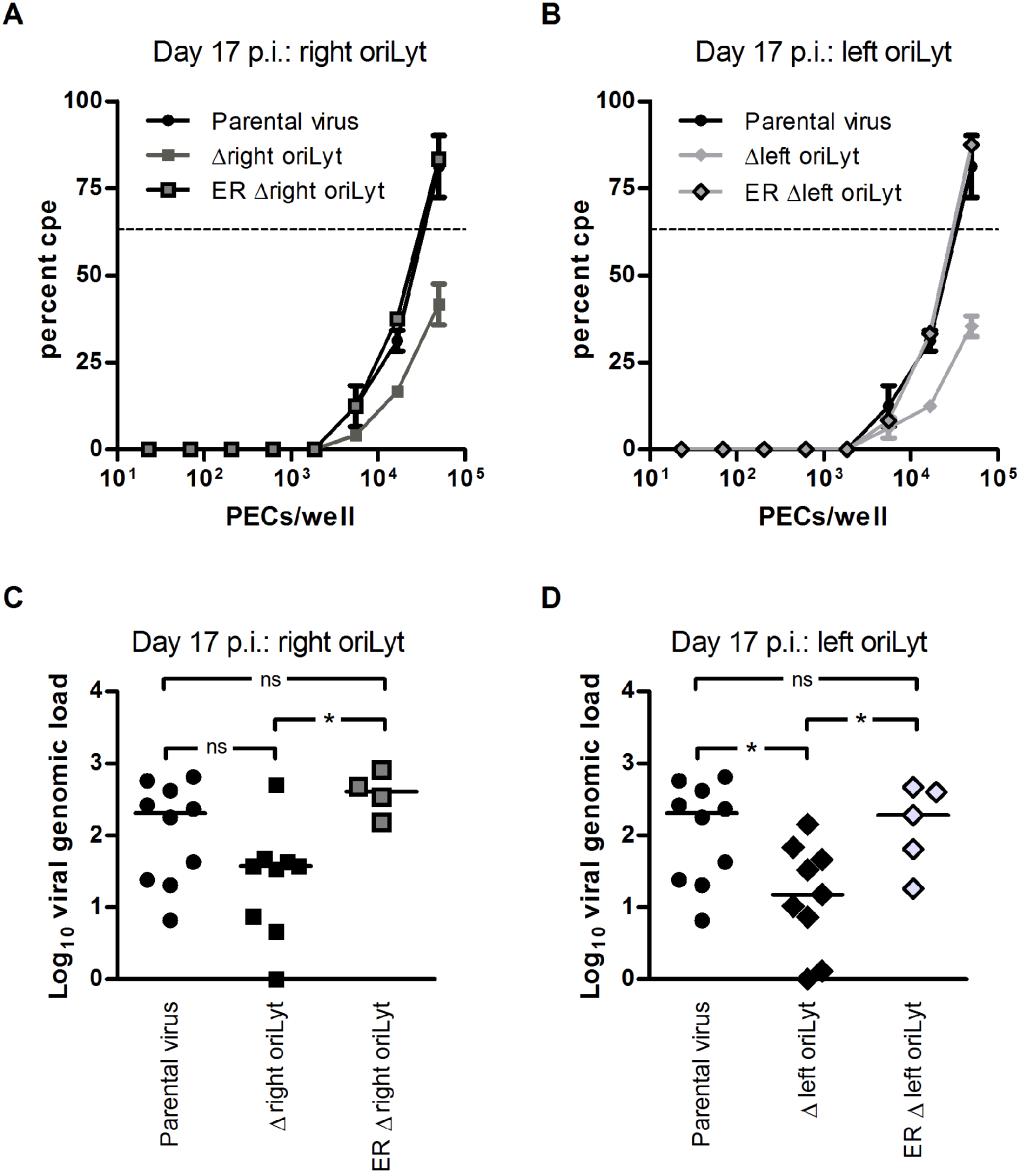
Latent infection in PECs after i.p. inoculation. C57BL/6 mice were inoculated i.p. with 1x 10^4^ PFU of the indicated viruses. PECs were harvested at day 17 after infection to analyze latent infection in the ex vivo reactivation assay (A and B) or for DNA isolation and real-time PCR analysis of the viral genomic load (C and D). Data for parental virus and virus mutants are obtained from two independent experiments, each with five mice per group. Data for the ectopic revertants (ER Δright oriLyt and ER Δleft oriLyt) are obtained from a single experiment with five mice per group. For the ex vivo reactivation assay, cells from five mice per group were pooled in each experiment and data shown are the means ± SEM (parental virus or virus mutants) or values from a single experiment (ectopic revertants). The dashed line indicates the point of 63.2% Poisson distribution, determined by nonlinear regression, which was used to calculate the frequency of cells reactivating lytic replication. In panels C and D, each symbol represents an individual mouse, and the bars represent the median value. Asterisks indicate a statistically significant difference between the groups (* P < 0.05).

The route of inoculation determines, at least to a certain extent, which cell types are initially infected and how viruses subsequently spread. Consequently, the requirements for oriLyts might differ between various routes of inoculation. Another site of MHV-68 latency is the peritoneum, where macrophages are the major cell type harbouring latent MHV-68 [17]. Thus, we also investigated latency establishment and reactivation from latency in peritoneal exudate cells (PECs) after intraperitoneal (i.p.) inoculation. C57BL/6 mice were inoculated i.p., and ex vivo reactivation and viral genomic load of latently infected PECs were determined 17 days p.i. The frequency of reactivating cells was lower in both groups infected with mutant virus compared to parental virus (Fig 4A and 4B; 1 in 34114 for parental virus, 1 in 145053 for the Δright oriLyt mutant, and 1 in 198144 for the Δleft oriLyt mutant). The viral copy number found in PECs of mice infected with the Δright oriLyt mutant was reduced only by trend but did not reach statistical significance (Fig 4C). In contrast, a significantly lower viral copy number was found in PECs of mice infected with the Δleft oriLyt mutant when compared to mice infected with parental virus (Fig 4D). Ex vivo reactivation and viral copy numbers after infection with the ectopic revertants were not significantly reduced when compared to parental virus (Fig 4A–4D). Thus, our results regarding viral latency after i.p. inoculation demonstrate that the deletion of the right or the left oriLyt influences latency establishment in PECs, and that differences regarding the need for two oriLyts exist depending on the compartment and the route of inoculation. Table B in S1 Text provides a schematic summary of all results obtained in vivo, indicating that, as in vitro, specific differences between the two oriLyt mutants also exist in vivo.

### Identification of proteins associated with the right oriLyt

Since we observed that the loss of one oriLyt can obviously be completely compensated by the other oriLyt in some but not in all cell types, we hypothesized that two oriLyts are necessary to guarantee optimal fitness throughout the viral life cycle in all cell types which are encountered during the course of infection. One way to achieve this goal might be by differential interaction of either oriLyt with cell-type specific factors which might impose either activating or inhibitory effects. To test this hypothesis, we chose to investigate the right oriLyt. To identify proteins that might interact with the right oriLyt in one cell line but not in the other, we selected TCMK-1, a cell line in which we had observed reduced growth of the Δleft oriLyt mutant (contains only the right oriLyt), and as control NIH 3T3, a cell line in which the Δleft oriLyt mutant grew like parental virus (Table A in S1 Text). Proteins bound to the right oriLyt were identified by a modification of the DNA-affinity purification method described previously by Wang et al. [6]. Using this method, 193 proteins could be identified which were exclusively detected in extracts from TCMK-1 cells and 37 proteins in extracts from NIH 3T3 cells (Table 1). Two candidate proteins, Hexim1, found in TCMK-1 cells only, and Rbbp4, found in NIH 3T3 cells only, were chosen for further investigation (Fig C in S1 Text and Table C in S1 Text). A list of all proteins which were identified by nanoHPLC-ESI-MS/MS is provided in S1 Table.

**Table 1:** Proteins associated with the right oriLyt as identified by DNA-affinity purification and mass spectrometry analysis^a^.

found in…	number of proteins	examples
Both cell lines	162	**DNA topoisomerase I**
		**X-ray repair cross-complementing protein 5 (Ku86)**
		**Poly [ADP-ribose] polymerase 1 (PARP1)**
		**Heterogeneous nuclear ribonucleoprotein A3 (hnRNP A3)**
TCMK-1	193	**Heterogeneous nuclear ribonucleoprotein K (hnRNP K)**
		Hexamethylene bis-acetamide-inducible protein 1 (HEXIM1)
		Cyclin-dependent kinase 9 (CDK9)
		Polycomb protein Suz12
NIH 3T3	37	**Heterogeneous nuclear ribonucleoprotein U (SAF-A)**
		Retinoblastoma-binding protein 4 (Rbbp4)
		7SK snRNA methylphosphate capping enzyme (MePCE)
		ASH2-like protein (ASH2L)

^a^ Proteins highlighted in bold letters were previously shown also to interact with the oriLyt of KSHV by Wang et al. [6].

**Fig 5:**
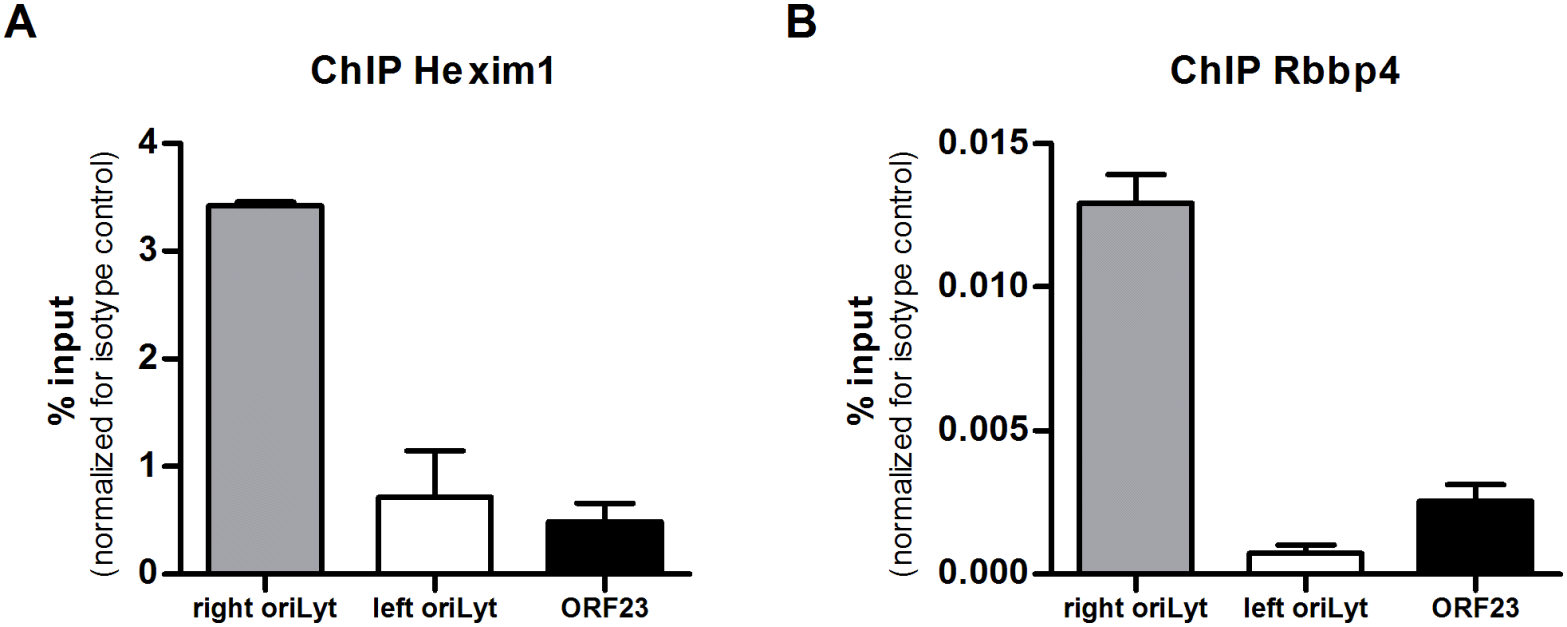
Confirmation of results from DNA-affinity purification and MassSpec. Formaldehyde cross-linking ChIP assays were performed to ensure that Hexim1 and Rbbp4 are associated with DNA of the right oriLyt of MHV-68. The immunoprecipitates isolated from TCMK-1 cells by a specific antibody against Hexim1 (A) or from NIH 3T3 cells by a specific antibody against Rbbp4 (B) were analyzed by quantitative PCR with primer pairs designed to amplify a part of the DNA sequence of the right oriLyt and the left oriLyt, respectively, or by a primer pair amplifying a non-relevant sequence located in the viral ORF23.

Since the MS data were suggestive of cell line specificity, we wanted to confirm the association of Hexim1 and Rbbp4 with the right oriLyt by additional approaches. First, formaldehyde cross-linking chromatin immunoprecipitation (ChIP) assays were performed. Chromatin from TCMK-1 and NIH 3T3 cells, respectively, infected with MHV-68, was immunoprecipitated with specific antibodies against Hexim1 or Rbbp4. Isotype matched antibodies were used as a control. Quantification of the precipitated protein-bound DNA by qPCR showed that the DNA of the right oriLyt could be enriched by precipitation with a Hexim1- or a Rbbp4-specific antibody while DNA of the left oriLyt or DNA of an unrelated genomic region (ORF23), that was used as a negative control, could not be enriched (Fig 5A and 5B). Second, Western Blots for Hexim1 and Rbbp4 with samples from both TCMK-1 and NIH 3T3 cells after DNA-affinity purification were performed (Fig D in S1 Text). Hexim1 was only detected in samples from TCMK-1 cells purified with the specific oriLyt DNA but not in NIH 3T3 cells, whereas Rbbp4 was only found in DNA-affinity purified samples from NIH 3T3 cells. As a control, Topoisomerase I could be detected in samples from both cell lines. No bands were found in any sample purified with unspecific control DNA.

### Hexim1 affects lytic growth of the Δleft oriLyt mutant

**Fig 6:**
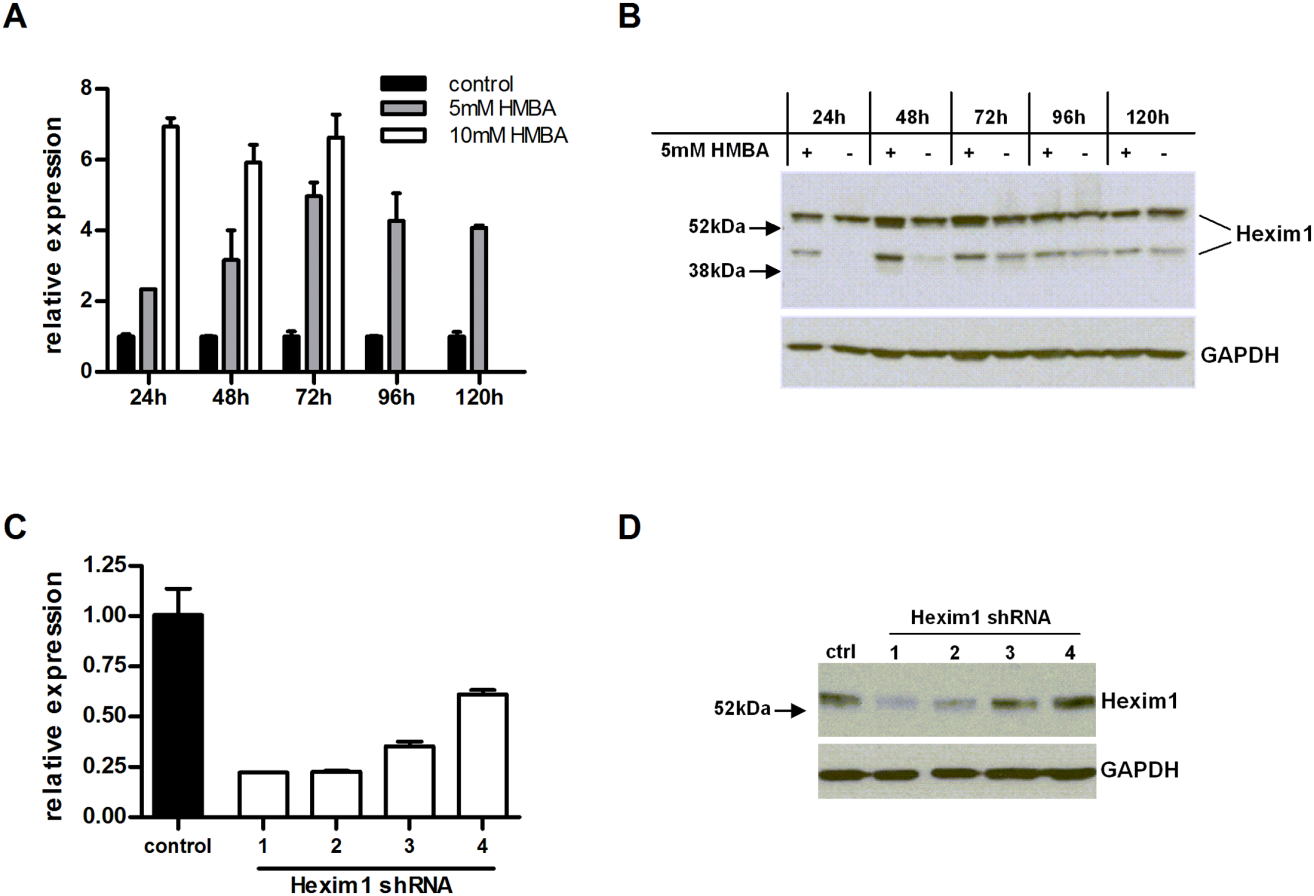
Confirmation of up- or downregulation of Hexim1. Upregulation of Hexim1 was accomplished by stimulation of NIH 3T3 cells with HMBA, and the expression of Hexim1 was analyzed by quantitative PCR on mRNA level (A) and by Western Blot on protein level (B). In addition to the predicted 54 kDa band, a second band at approximately 40 kDa was detected by Western Blot with lysates from NIH 3T3 cells, probably representing a modified form of the protein. To downregulate Hexim1, stable cell lines of TCMK-1 cells expressing shRNA specific for Hexim1 were generated. Downregulation of Hexim1 in these cell lines was confirmed by quantitative PCR (C) and by Western Blot (D). The shRNAs 1 and 2 proved to be most effective regarding downregulation of Hexim1.

**Fig 7:**
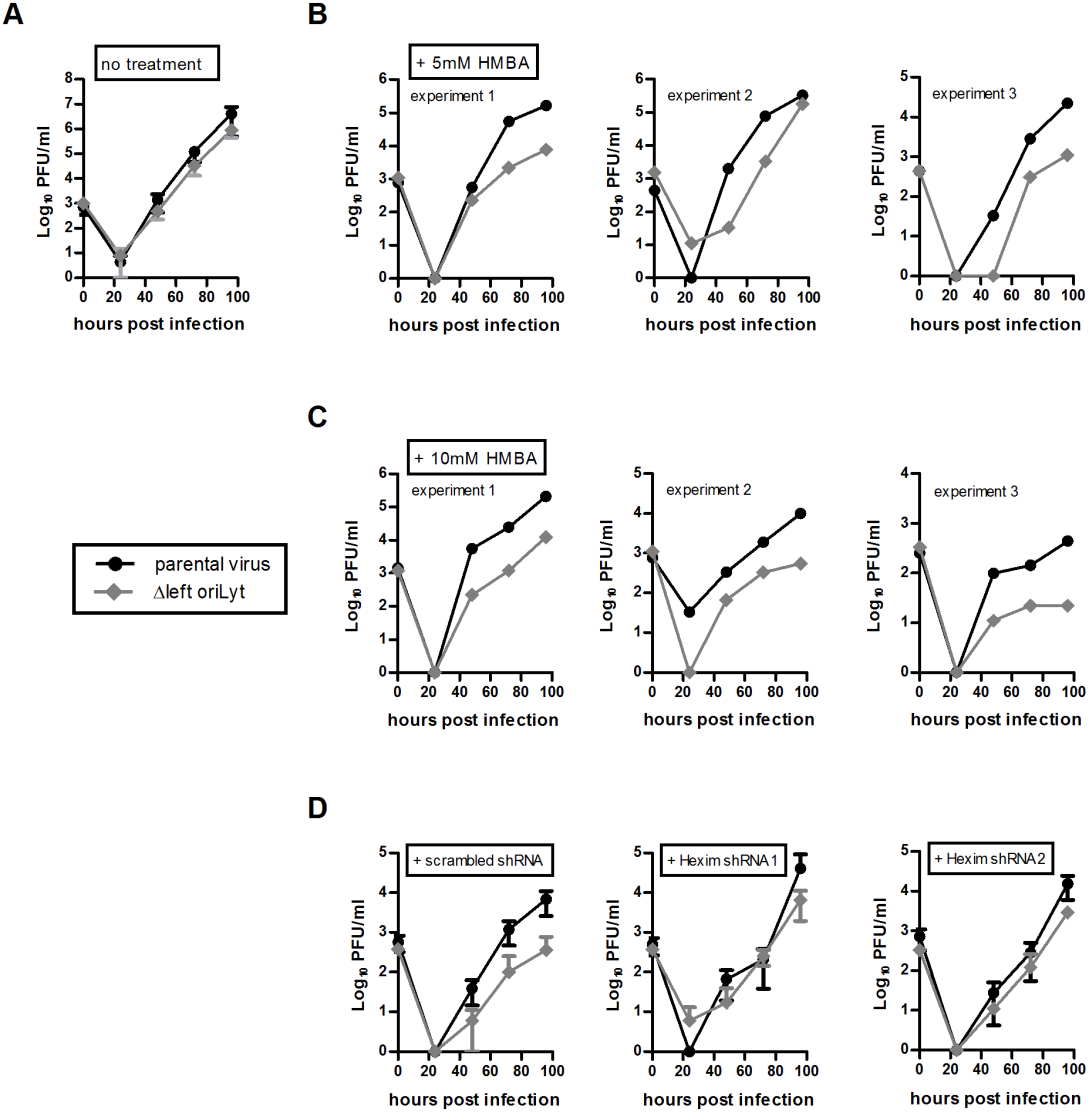
Virus replication in vitro after upregulation or downregulation of Hexim1. To upregulate Hexim1, NIH 3T3 cells were plated and after 4 hours stimulated with 5 mM or 10 mM HMBA or left untreated. 24 hours later, cells were infected with the indicated viruses at an MOI of 0.01 for 1 hour. After removing the inoculum, cells were incubated with fresh medium without HMBA (A), with 5 mM HMBA (B) or with 10 mM HMBA (C) at 37°C and 5% CO_2_ until the supernatants together with the cells were harvested at different time points after infection. Virus titers were determined by plaque assay. For the “no treatment” control, the means ± SEM of duplicates from three independent experiments are shown. Data shown for the HMBA treated cells are from three independent experiments showing each single experiment. To downregulate Hexim1, TCMK-1 cells were stable transfected with two different shRNAs specific for Hexim1 or with a scrambled control shRNA, respectively (D). Cells were plated and after 24 hours infected with the indicated viruses at an MOI of 0.01 for 1 hour. After removing the inoculum, cells were incubated with fresh medium at 37°C and 5% CO_2_ until the supernatants together with the cells were harvested at different time points after infection. Virus titers were determined by plaque assay. For the “scrambled shRNA” control, the means ± SEM of duplicates from two independent experiments are shown. For the shRNAs specific for Hexim1, the means ± SD of two independent experiments each are shown.

Hexim1 was found to be associated with the DNA of the right oriLyt in TCMK-1 cells but not in NIH 3T3 cells. Since the mutant containing only the right oriLyt showed reduced growth in TCMK-1 cells but not in NIH 3T3 cells, we hypothesized that in TCMK-1 cells, Hexim1 exerts an inhibitory effect on lytic replication originating at the right oriLyt. This inhibitory effect is normally compensated by the left oriLyt but this is not the case when the left oriLyt is absent. Consequently, downregulation of Hexim1 in TCMK-1 cells should reverse the growth deficit of the mutant containing only the right oriLyt, while forced overexpression of Hexim1 in NIH 3T3 cells might result in a growth deficit of this mutant. To prove these hypotheses, we first upregulated Hexim1 in NIH 3T3 cells by treatment with the chemical compound Hexamethylene bis-acetamide (HMBA) which is known to induce expression of Hexim1 [24,25]. Two different concentrations of HMBA were used, and upregulation of Hexim1 was confirmed by RT-PCR and Western Blot (Fig 6A and 6B). Multistep growth curves were performed with HMBA-treated cells and the growth of parental virus and the mutant containing only the right oriLyt was tested in parallel with or without HMBA-treatment. The viral titers of the mutant containing only the right oriLyt were comparable to parental virus in untreated cells, but treatment with HMBA (both at 5 or 10 mM) resulted in viral titers of about one order of magnitude lower than in cells infected with parental virus (Fig 7A–7C), indicating that Hexim1 in fact reduces virus replication originating at the right oriLyt. This inhibitory effect was specific for the right oriLyt since treatment with 10 mM HMBA did not significantly affect the growth of the mutant containing only the left oriLyt (Fig E in S1 Text). In a second set of experiments, expression of Hexim1 was downregulated in TCMK-1 cells. For this purpose, stable cell lines expressing shRNAs specific for Hexim1 were generated. Downregulation was confirmed on mRNA and protein level (Fig 6C and 6D). Two cell lines with the most prominent reduction of Hexim1 expression were chosen and multistep growth curves were performed. Again, lytic growth of the parental virus and the mutant containing only the right oriLyt was tested in parallel in the same cell lines. As a control, a cell line stably expressing a scrambled shRNA was used. Consistent with our previous results (Fig 1D), the mutant containing only the right oriLyt showed reduced growth in the control cell line expressing a scrambled shRNA when compared to the parental virus. However, in the cell lines expressing shRNAs specific for Hexim1, no or only a marginal reduction in lytic virus growth of the mutant containing only the right oriLyt was observed when compared to parental virus (Fig 7D). We conclude from these results that Hexim1 inhibits lytic replication originating at the right oriLyt but this is normally compensated by the presence of the left oriLyt.

### Rbbp4 differentially affects lytic growth of oriLyt mutants

**Fig 8:**
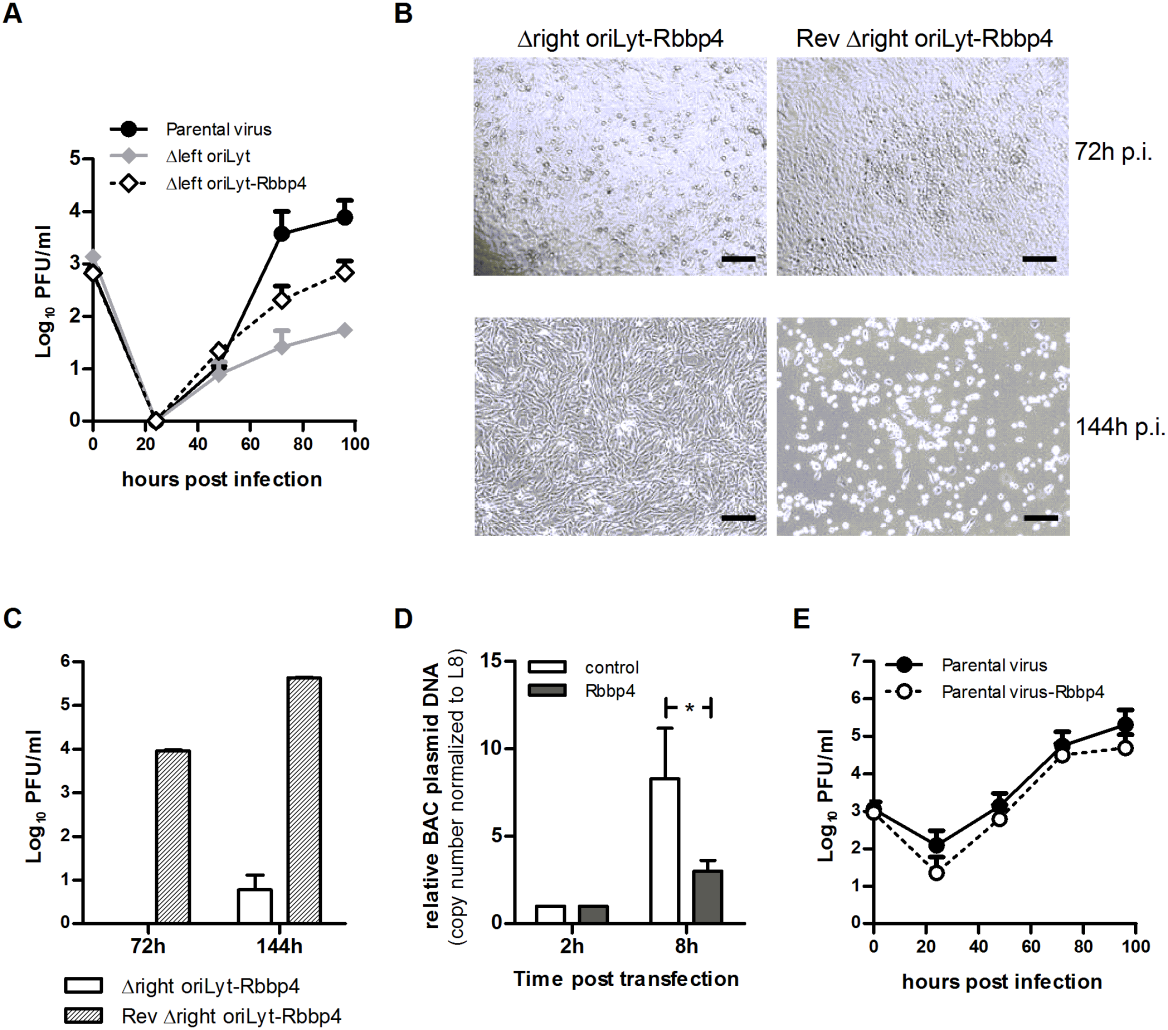
Analysis of the influence of Rbbp4 on lytic virus replication. To analyze lytic growth of recombinant viruses expressing Rbbp4, TCMK-1 cells were plated and after 24 hours infected with the indicated viruses at an MOI of 0.01 for 1 hour. After removing the inoculum, cells were incubated with fresh medium at 37°C and 5% CO_2_ until the supernatants together with the cells were harvested at the indicated time points after infection. Virus titers were determined by plaque assay. The means + SD of three independent experiments are shown (A and E). BHK-21 cells were plated and after 24 hours transfected with 2 μg of the indicated BAC plasmid DNA. Plaque development and cytopathic effect (virus reconstitution) were monitored by light microscopy, and photomicrographs were taken at the indicated time points. Scale bar, 500 μm (B). Cell free supernatants from (B) were harvested and virus titers were determined by plaque assay. Data shown are means + SD from duplicate determinations (C). BAC plasmid DNA replication was measured by real-time PCR analysis after co-transfection of a BAC plasmid lacking the right oriLyt with an Rbbp4-expression plasmid or a control plasmid. Data shown are means + SD from three independent experiments (D).

Rbbp4 was found to be associated with the DNA of the right oriLyt in NIH 3T3 cells but not in TCMK-1 cells (Table 1). The mutant containing only the right oriLyt showed reduced growth in TCMK-1 cells but not in NIH 3T3 cells (Table A in S1 Text). Thus, we hypothesized that Rbbp4 supports lytic replication originating at the right oriLyt. Consequently, forced overexpression of Rbbp4 in TCMK-1 cells should, at least partially, release the growth deficit of the mutant containing only the right oriLyt also in this cell line. To prove this hypothesis, we inserted an Rbbp4 expression cassette in the mutant containing only the right oriLyt, resulting in the recombinant virus Δleft oriLyt-Rbbp4. Thus, infection with this mutant should lead to the expression of Rbbp4 in infected cells. This was confirmed by Western Blot (Fig F in S1 Text). Multistep growth curves were performed and the growth of parental virus, Δleft oriLyt mutant (containing only the right oriLyt) and Δleft oriLyt-Rbbp4 virus was tested in parallel. Expression of Rbbp4 by the Δleft oriLyt-Rbbp4 virus partially reversed the growth deficit of the Δleft oriLyt mutant (Fig 8A), indicating that Rbbp4 in fact supports virus replication originating at the right oriLyt. In contrast to the mutant containing only the right oriLyt, the mutant containing only the left oriLyt showed reduced growth in NIH 3T3 cells but not in TCMK-1 cells (Table A in S1 Text). Thus, we hypothesized that Rbbp4 exerts an inhibitory effect on lytic replication originating at the left oriLyt. If so, forced overexpression of Rbbp4 should inhibit lytic replication of the mutant containing only the left oriLyt even further. Moreover, neither the inhibitory nor the supporting effect of Rbbp4 overexpression should become apparent in the parental wildtype virus since both effects are compensated by the simultaneous presence of both the right and the left oriLyt. To prove these hypotheses, we also inserted the Rbbp4 expression cassette both in the mutant containing only the left oriLyt and in the parental virus, resulting in the recombinant viruses Δright oriLyt-Rbbp4 and parental virus-Rbbp4, respectively. Again, infection with these recombinant viruses resulted in expression of Rbbp4 in infected cells which was confirmed by Western Blot (Fig F in S1 Text). Consistent with our hypothesis, overexpression of Rbbp4 exerted an inhibitory effect on the mutant containing only the left oriLyt, notably to a very strong extent. In fact, it was not possible to reconstitute considerable amounts of infectious virus after transfection of cells with the respective BAC DNA (Fig 8B, left panel, and Fig 8C). Since there was such a strong effect, we attempted to exclude any unwanted side effects which might have occurred during the construction of the recombinant virus Δright oriLyt-Rbbp4. To this end, we re-inserted the right oriLyt into the Δright oriLyt-Rbbp4 virus, resulting in the recombinant virus Rev Δright oriLyt-Rbbp4. Transfection of the respective BAC DNA readily resulted in virus reconstitution and production of infectious virus (Fig 8B, right panel, and Fig 8C), thus clearly demonstrating the specificity of the Rbbp4 inhibitory effect on the left oriLyt. To get a first insight whether the observed inhibitory effect of Rbbp4 on the left oriLyt occurs at the level of DNA replication, we performed a modified plasmid replication assay using the BAC plasmid of the Δright oriLyt mutant (containing only the left oriLyt). To this end, we co-transfected the BAC plasmid DNA with either an Rbbp4-expression plasmid or an appropriate control plasmid (expressing gfp), and measured the replication of the BAC plasmid DNA by real-time PCR eight hours after transfection. Co-transfection with the Rbbp4-expression plasmid significantly inhibited the replication of the BAC plasmid DNA, when compared to the co-transfection with the control plasmid (Fig 8D). Finally, consistent with our hypothesis, the growth of the parental virus was not affected by overexpression of Rbbp4 (Fig 8E). We conclude from these results that Rbbp4 may exert specific effects on each oriLyt and that wildtype virus is able to circumvent potential negative effects by the presence of more than one oriLyt.

## Discussion

Several herpesviruses including EBV and KSHV have two oriLyts but the need for more than one oriLyt has never been defined [26]. In clinical EBV isolates, usually both oriLyts are found but there are also laboratory strains, such as B95-8, which have only one oriLyt and still can replicate lytically, at least in vitro [8]. The standard assay for studying oriLyt-dependent replication is the so called "transient in vitro plasmid replication assay". In this assay, a plasmid carrying the oriLyt sequence is transfected into cells and the trans-factors needed are provided by co-transfection or co-infection. Then, the replication ability of the plasmid is determined [9]. This assay which examines the role of oriLyts as isolated DNA sequences cloned into a plasmid provides important information but says little about the role of oriLyts in the context of an infection [27]. Therefore, recombinant BACs are increasingly used to investigate the function of the oriLyts. Using an EBV BAC, for example, it was shown that the BZLF1 oriLyt binding sites (ZRE) are important but not essential for lytic replication [27]. The analysis of recombinant KSHV BACs revealed that KSHV can replicate lytically in vitro with only one oriLyt. Interestingly, deletion of the right oriLyt had no influence on replication, whereas deletion of the left oriLyt led to the loss of replicative capacity [28]. In previous work, using recombinant MHV-68 generated by BAC-technology, we could show that the absence of one oriLyt was well tolerated regarding in vitro replication in NIH 3T3 cells [18] whereas in vivo, a significant reduction of viral fitness was observed [20]. Viruses lacking both oriLyts could not replicate at all [18].

Here, using recombinant, BAC-based MHV-68, we systematically addressed the question why some herpesviruses need more than one oriLyt. Our central working hypothesis was that the presence of two oriLyts provides optimal fitness to efficiently establish infection in different cell or tissue types and during different phases of the viral life cycle by enabling interaction with cell-type specific cellular proteins which might impose either activating or inhibitory effects. Indeed, loss of either of the two oriLyts was well tolerated in some cell types but not in others in vitro. Similarly, in vivo, loss of either of the two oriLyts resulted in different effects on the viral life cycle dependent on the organ and the route of inoculation. For example, both oriLyt mutants showed reduced lytic replication in vitro in the alveolar epithelial cell line LA-4 (Table A in S1 Text), with a tendency of a stronger reduction of the Δright oriLyt mutant. The same phenotype was observed during lytic replication in vivo in the lung (Fig 2) which mainly takes place in alveolar epithelial cells. Thus, both the in vitro and the in vivo data were consistent and in support of our hypothesis. Identification of oriLyt-bound cellular proteins by DNA-affinity purification and mass spectrometry revealed that, depending on the cell line, a different repertoire of proteins seemed to interact with the respective oriLyt or the associated replication complex. We selected two of the differentially oriLyt-interacting cellular proteins, Hexim1 and Rbbp4, for further investigation. In a recently performed extensive mutagenesis screen in haploid human cells, Hexim1 was identified as a non-critical gene while Rbbp4 was found to be a core essential gene that is required for cell survival [29].

In the absence of the left oriLyt, Hexim1 exerted an inhibitory effect on lytic replication in TCMK-1 but not in NIH 3T3 cells. Hexim1 is an inhibitor of positive transcription elongation factor b (P-TEFb) which plays a key role in regulation of RNA polymerase II elongation [30]. P-TEFb consists of cyclin-dependent kinase 9 (cdk9) and cyclin 1 [30]. In conjunction with 7SK non-coding RNA, Hexim1 inhibits the kinase activity of cdk9 [30]. Interestingly, it has been shown that inhibition of cdk9 by overexpression of Hexim1 resulted in decreased viral yields during herpes simplex virus 1 infection [31]. We demonstrated that upregulation of Hexim1 in NIH 3T3 cells, a cell line where it was not found to be associated with the right oriLyt, inhibited virus replication of the mutant lacking the left oriLyt. Since Hexim1 is present in extracts of NIH 3T3 cells but could not be enriched by DNA affinity purification with DNA of the right oriLyt (Fig D in S1 Text), we assume that Hexim1 does not directly bind to the DNA of the right oriLyt but rather via one or more additional interaction partners which might be present in TCMK-1 but not in NIH 3T3 cells. It is currently not clear how forced overexpression of Hexim1 can exert the observed effect on lytic replication at the right oriLyt. One possible explanation might be that, by the high abundance of Hexim1 after HMBA-induced upregulation, the normally strict need for an additional interaction partner is circumvented. Alternatively, HMBA might also induce or up-regulate potential interaction partners. Another explanation might be that the effect is mediated by an additional protein form of Hexim1 which we detected after treatment of NIH 3T3 cells with HMBA (Fig 6). The identity and function of this form has not been described yet. Downregulation of Hexim1 in TCMK-1, a cell line where it was found to be associated with the right oriLyt, enhanced replication of the mutant lacking the left oriLyt. Thus, overexpression and downregulation of Hexim1 either reduced or enhanced, depending on the cell line, the replication of the mutant lacking the left oriLyt, indicating that Hexim1 is a rate limiting cellular protein in a situation where only one oriLyt is present. However, the virus can overcome this hurdle by the presence of two oriLyts, indicating that two oriLyts are of advantage for optimal virus fitness. How Hexim1 is functioning in the context of oriLyt-dependent DNA replication is not known at the moment but we can envisage several scenarios: i) Hexim1 has additional, so far unknown functions in DNA-replication. ii) By its interference with transcription, it might inhibit the generation of various RNA molecules which might aid in lytic DNA replication. iii) Since it can bind RNA, it might simply sequester RNAs important for lytic DNA replication. The latter two possibilities are particularly attractive since it has been shown that a GC-rich oriLyt transcript is an important component of the EBV oriLyt [32]. Similarly, a tight coupling of DNA replication and transcription was shown for KSHV: oriLyt-dependent DNA replication was inhibited when RNA transcription was prematurely terminated [33,34].

The cellular protein Rbbp4 supported lytic replication originating at the right oriLyt while it strongly inhibited lytic replication originating at the left oriLyt. We could show that the inhibitory effect of Rbbp4 on the left oriLyt also occurs at the level of DNA replication, however, this does not rule out additional effects on other steps of the viral life cycle. While we could demonstrate an interaction of Rbbp4 with the right oriLyt, we did not find a direct interaction of Rbbp4 with the left oriLyt. Thus, it is currently not clear how forced overexpression of Rbbp4 can exert the observed inhibitory effect on lytic replication at the left oriLyt. Nevertheless, it seems that Rbbp4, like Hexim1, is a rate limiting cellular protein in situations where only one oriLyt is present. Again, the virus can overcome potential negative effects by the presence of two oriLyts. Rbbp4, also known as RbAp48 or NURF55, is a component of several chromatin-related complexes, for example NuRD (nucleosome remodeling histone deacetylase complex) and CAF-1 (chromatin assembly factor-1) [35]. The function of CAF-1 is known to be tightly associated with DNA replication [35]. Interestingly, Rbbp4, most likely as a component of a functional NuRD complex, has been shown to be required for efficient replication of human cytomegalovirus (HCMV) [36]. Thus, it is easily conceivable that Rbbp4 might be involved in oriLyt-dependent DNA replication.

Taken together, our data suggest that the presence of multiple oriLyts enables γHV to efficiently deal with the variety of conditions which they encounter during the viral life cycle, for example by facilitating interaction with cell-type specific proteins.

## Materials and Methods

### Cell lines

All cell lines used in this study were cultured under standard conditions and have been described before [37–39] or are commercially available (American Type Culture Collection [ATCC] or Leibniz Institute DSMZ—German Collection of Microorganisms and Cell Cultures [DSMZ]). More details are provided in the Supplementary Information.

### Plasmid construction

For this study, we constructed eight recombinant MHV-68 (schematically depicted in Fig A in S1 Text): 1) The mutant virus Δleft oriLyt, lacking the essential part of the left oriLyt [19]. 2) A revertant of the Δleft oriLyt mutant with an ectopic insertion of the left oriLyt (ER Δleft oriLyt). 3) The mutant virus Δright oriLyt with a deletion of the essential part of the right oriLyt. This mutant has been described previously [18]. 4) A revertant of the Δright oriLyt mutant with an ectopic insertion of the right oriLyt (ER Δright oriLyt). 5) Three recombinant MHV-68 expressing Rbbp4 from an intergenic expression cassette: Parental virus-Rbbp4, Δleft oriLyt-Rbbp4 and Δright oriLyt-Rbbp4. 6) A revertant of the Δright oriLyt-Rbbp4. Details on the construction of all recombinant MHV-68 used in this study can be found in the Supplementary Information.

### Reconstitution and characterization of recombinant MHV-68

To reconstitute recombinant MHV-68, BHK-21 cells were transfected with 2 μg of BAC MHV-68 DNA using X-treme GENE HP DNA Transfection Reagent (Roche, Mannheim, Germany). When cells showed a total cytopathic effect (CPE), an aliquot of the supernatant was used to infect Ref-Cre cells carrying the Cre recombinase to remove the BAC-cassette including the GFP sequence. Removal of the BAC-cassette was done for all viruses to be used in vivo. BAC-cassette free viruses were identified by a limiting dilution assay on BHK-21 cells performed in a 96 well plate. All recombinant viruses were grown and titrated on BHK-21 cells as previously described [37], and were characterized by restriction enzyme and Southern blot analysis and by sequencing across the mutated regions.

### In vitro experiments

To test in vitro growth of the virus mutants, cells of different type and origin were infected with a MOI of 0.01 (or 1 for MH-S macrophages) for one hour. After removing the inoculum, cells were incubated with fresh medium at 37°C and 5% CO_2_ until the supernatants together with the cells were harvested at different time points after infection. Virus titers were determined by plaque assay.

### In vivo experiments

Female C57BL/6 mice (6–8 weeks old) were purchased from Charles River Laboratories (Sulzfeld, Germany) and housed in individually ventilated cages (IVC) during the MHV-68 infection period. To characterize the recombinant MHV-68 in vivo, mice were inoculated i.n. with 1x 10^3^ PFU or i.p. with 1x 10^4^ PFU. Prior to i.n. inoculation, mice were anesthetized with ketamine and xylazine. To determine virus titers, organs were harvested at the indicated time points after infection and homogenized by using the FASTPREP-24 instrument (MP Biomedicals, Heidelberg, Germany). After two times freezing and thawing the homogenates, plaque assays were performed with 10-fold dilutions of the supernatants on BHK-21 cells. For determination of spleen weight, frequency of virus reactivation and genomic load, spleens were harvested at the indicated time points after infection.

### Ethics statement

All animal experiments were in compliance with the German Animal Welfare Act (German Federal Law §8 Abs. 1 TierSchG), and the protocol was approved by the local Animal Care and Use Committee (District Government of Upper Bavaria; permit number 124/08).

### Limiting dilution reactivation assay

To determine the frequency of cells carrying virus reactivating from latency, threefold dilutions of splenocytes or PECs were plated onto NIH 3T3 cells as described previously [40]. Frequencies of reactivating cells were calculated on the basis of the Poisson distribution by determining the cell number at which 63.2% of the wells scored positive for CPE.

### Measurement of latent viral load by real time PCR

Viral load in splenocytes or PECs of infected mice was determined by quantitative real-time PCR using the ABI 7300 Real Time PCR System (Applied Biosystems, Foster City, CA) as described previously [20].

### DNA-affinity purification

Proteins bound to the right oriLyt were purified using a modified method described earlier by Wang et al. [6]. A biotinylated DNA fragment spanning the minimal region of the right oriLyt plus a few extra nucleotides on both sides (genome coordinates 100.018–102.031) was amplified by PCR with MHV-68 DNA as a template. The primers for the right oriLyt were ori_right.for (5′-AGCGAGGGAGCGGGCTGC-3′) and ori_right_Bio.rev (5′-biotin-CCTACGTCATCAAGCAGCGACG-3′). An unspecific PCR-product amplified from the Kanamycin resistance cassette of the plasmid pCP15 was used as a control. The primers for this control were pCP15Kan_Bio.for (5′-biotin-CCAGGGTTTTCCCAGTCACGACGT-3′) and pCP15Kan.rev (5′-CACAGGAAACAGCTATGACCATGA-3′). The resultant biotinylated PCR fragments were diluted in buffer 1 (20 mM HEPES, pH 7.9, 20% glycerol, 0.2 mM EDTA, 1 mM dithiothreitol (DTT), 0.05% NP-40, 15 mM MgCl_2_, 75 μg/ml salmon sperm DNA) and mixed with nuclear extracts prepared from cells infected with recombinant MHV-68 at an MOI of 0.1 for 48h. In each reaction mixture, 2/3 volume of biotinylated PCR-product was mixed with 1/3 volume of the nuclear extract in buffer 2 (20 mM HEPES, pH 7.9, 25% glycerol, 0.2 mM EDTA, 0.42 M NaCl, 1 mM DTT, 0.05% NP-40, and a protease inhibitor tablet) and incubated for 10 min at room temperature. Streptavidin MicroBeads (Miltenyi Biotec, Bergisch Gladbach, Germany) were added to each sample and incubated at room temperature for 5 min. The labeled samples were applied to a MACS column placed in a MACS separator and the column was washed three times in D150 buffer (20 mM HEPES, pH 7.9, 20% glycerol, 0.2 mM EDTA, 150 mM KCl, 1 mM DTT, 0.05% NP-40) and three times in D300 buffer (same as D150 buffer, except the KCl concentration was increased to 300mM). The bound material was pre-incubated with 25μl buffer D500 (KCl 500 mM) for 10 min at room temperature and then eluted with 50μl buffer D500. DNA affinity-purified proteins were resolved on 10% Mini-PROTEAN TGX gels (BioRad, Munich, Germany) and stained with FireSilver staining kit (Proteome Factory AG, Berlin, Germany). The protein bands were excised and subjected to mass spectrometric analyses.

### Protein identification by nanoHPLC-ESI-MS/MS

Protein identification using nanoHPLC-ESI-MS/MS was performed by Proteome Factory (Proteome Factory AG, Berlin, Germany). Details can be found in the Supplementary Information.

### Chromatin ImmunoPrecipitation (ChIP)

ChIP was performed according to standard protocols and is described in detail in the Supplementary Information.

### Overexpression or knockdown of proteins

Overexpression of Hexim1 was accomplished by treatment with hexamethylene bis-acetamide (HMBA) which induces Hexim1 expression [24,25]. 5mM or 10mM HMBA (Sigma-Aldrich, Seelze, Germany) was added to NIH 3T3 cells for the indicated time periods. For downregulation of Hexim1, four different TCMK-1/shRNA cell lines were established. Each cell line was stably transfected with a plasmid vector expressing shRNA specific for Hexim1 (Genecopoeia, Rockville, MD). The 19mer sequences specific for Hexim1 were: shRNA1: 5’-GTTGTCCATGAAGAGCATA-3’; shRNA2: 5’-TTAAGCGGAGCTATAAGGT-3’; shRNA3: 5’-GTTTGCCTACCTTGGTAAG-3’; shRNA4: 5’-TGCAGCTATTCTCAATCTC-3’. As a control, shRNA with a scrambled sequence was used (Genecopoeia, Rockville, MD). Cells stably expressing shRNA were selected by using puromycin at a concentration of 5 μg/ml. Up- or downregulation of Hexim1 was determined by RT-PCR and Western Blot. Overexpression of Rbbp4 was accomplished by insertion of a Rbbp4 expression cassette in the respective viruses, and overexpression of Rbbp4 was determined by Western Blot of infected cells.

### Western blot

Overexpression/downregulation of selected proteins or presence of proteins in DNA-affinity purified samples was analyzed by Western Blot. For analysis of protein expression, cells were treated as specified and lysed in 2x Lämmli buffer. DNA affinity purified samples were prepared as described and mixed with an equal amount of 2x Lämmli buffer. The samples were separated by SDS-Page gel electrophoresis and transferred to nitrocellulose membrane. The membrane was blocked in 5% skim milk (for Rbbp4, Topoisomerase I, and GAPDH) or 5% skim milk plus 0,5% BSA (for Hexim1) for 1 hour at room temperature and then incubated with primary antibody in blocking solution at 4°C overnight. Primary antibodies used in this paper were: rabbit anti-Hexim1 (Abcam, Cambridge, UK; dilution 1:2000), rabbit anti-Rbbp4 (Novus Biologicals, Cambridge, UK; 1:10000), rabbit anti-Topoisomerase I (Abcam, Cambridge, UK; dilution 1:10000), and rabbit anti-GAPDH (Cell Signaling, Boston, MA; 1:2000). The membrane was washed and then incubated with horseradish peroxidase-conjugated donkey-anti-rabbit IgG (Amersham/GE Healthcare, Freiburg, Germany; 1:5000) for 1 hour at room temperature. The signal was detected by an enhanced chemiluminescence system (Pierce/Thermo Scientific, Rockford, IL) on X-ray film.

### RT-PCR

mRNA was isolated from cell lines expressing shRNAs or HMBA-stimulated cells using the RNeasy MiniKit (Qiagen, Hilden, Germany). Genomic DNA was removed with the TURBO DNA-free Kit (Ambion/Life Technologies, Darmstadt, Germany), and RNA was reverse-transcribed using the High Capacity cDNA Reverse Transcription Kit (Applied Biosystems, Foster City, CA). The cDNA was analyzed for the expression of Hexim1 and the ribosomal Protein L8 by real time quantitative PCR using the Taqman SYBR green PCR master mix (Applied Biosystems, Foster City, CA). For Hexim1, a commercially available primer set was used (Qiagen, Hilden, Germany). The following PCR primer set was used for L8: forward 5’-CAG TGA ATA TCG GCA ATG TTT TG-3’; reverse 5’-TTC ACT CGA GTC TTC TTG GTC TC-3’. The fold change in expression of each target mRNA relative to L8 was calculated based on the threshold cycle (Ct) as 2^-Δ (ΔCt)^, where ΔCt = Ct_Hexim_-Ct_L8_ and Δ(ΔCt) = ΔCt_treated_-ΔCt_control_.

### Quantification of BAC plasmid DNA replication

BHK-21 cells were co-transfected with 200ng BAC plasmid DNA of the Δright oriLyt mutant and 600ng of an Rbbp4-expression plasmid (OriGene Technologies, Rockville, MD, USA) using X-treme GENE HP DNA Transfection Reagent (Roche, Mannheim, Germany). Co-transfection of BAC plasmid DNA with a GFP-expression plasmid (OriGene Technologies, Rockville, MD, USA) was used as a control. Cells were harvested 2 hours (= input DNA) and 8 hours (= input + replicated DNA) after transfection. DNA was isolated and the BAC plasmid DNA copy number in the transfected cells was determined by quantitative real-time PCR using the ABI 7300 Real Time PCR System (Applied Biosystems, Foster City, CA) as described previously [20].

### Statistical methods

Datasets were tested for Gaussian distribution before statistical analysis by D’Agostino-Pearson omnibus K2 normality test using the GraphPad Prism software, vs5 (GraphPad Software, Inc., San Diego, CA, USA). Datasets that passed the normality test were analyzed by Student’s t-test. All other datasets were analyzed by Mann-Whitney U test. Results with a p-value < 0.05 were considered significant.

## Supporting Information

S1 Text Includes Supplementary Figures A-F, Supplementary Tables A-C, Supplementary Methods and Supplementary References. Figure A: Schematic diagram showing the structure of the recombinant viruses used in this study. Figure B: Replication of oriLyt mutants and corresponding ectopic revertants in vitro. Figure C: MS/MS spectra for selected peptides. Figure D: Confirmation of results from DNA affinity purification and MassSpec by Western Blot. Figure E: Replication of parental virus and Δright oriLyt mutant in vitro after upregulation of Hexim1. Figure F: Confirmation of overexpression of Rbbp4. Table A: Lytic growth of oriLyt mutants in vitro. Table B: Phenotype of oriLyt mutants in vivo. Table C: Peptides detected from selected proteins by MassSpec analysis.
(DOCX)

S1 Table Supplementary Table 1. List of all proteins which were identified by nanoHPLC-ESI-MS/MS.
(XLSX)

## References

[1] Roizman B, Knipe DM, Whitley RJ (2007) Herpes simplex viruses In: Knipe DM, Howley PM, Griffin DE, editors. Fields—Virology. Philadelphia: Lippincott Williams & Wilkins pp. 2501–2601.

[2] Mocarski J (2007) Comparative analysis of herpesvirus-common proteins In: Arvin AM, Campadelli-Fiume G, Mocarski ES, Moore PS, Roizman B et al, editors. Human Herpesviruses: Biology, Therapy, and Immunoprophylaxis. Cambridge: Cambridge University Press pp. 44. 21348071

[3] Pari GS, AuCoin D, Colletti K, Cei SA, Kirchoff V, Wong SW Identification of the rhesus macaque rhadinovirus lytic origin of DNA replication. J Virol. 2001; 75:11401–11407. 11689621 10.1128/JVI.75.23.11401-11407.2001 PMC114726

[4] Deng H, Chu JT, Park NH, Sun R Identification of cis sequences required for lytic DNA replication and packaging of murine gammaherpesvirus 68. J Virol. 2004; 78:9123–9131. 15308708 10.1128/JVI.78.17.9123-9131.2004 PMC506910

[5] AuCoin DP, Colletti KS, Cei SA, Papouskova I, Tarrant M, Pari GS Amplification of the Kaposi's sarcoma-associated herpesvirus/human herpesvirus 8 lytic origin of DNA replication is dependent upon a cis-acting AT-rich region and an ORF50 response element and the trans-acting factors ORF50 (K-Rta) and K8 (K-bZIP). Virology. 2004; 318:542–555. 14972523 10.1016/j.virol.2003.10.016

[6] Wang Y, Li H, Tang Q, Maul GG, Yuan Y Kaposi's sarcoma-associated herpesvirus ori-Lyt-dependent DNA replication: involvement of host cellular factors. J Virol. 2008; 82:2867–2882. 10.1128/JVI.01319-07 18199640 PMC2259006

[7] Gonzalez-Molleda L, Wang Y, Yuan Y Potent Antiviral Activity of Topoisomerase I and II Inhibitors against Kaposi's Sarcoma-Associated Herpesvirus. Antimicrob Agents Chemother. 2012; 56:893–902. 10.1128/AAC.05274-11 22106228 PMC3264263

[8] Hammerschmidt W, Sugden B Identification and characterization of oriLyt, a lytic origin of DNA replication of Epstein-Barr virus. Cell. 1988; 55:427–433. 2846181 10.1016/0092-8674(88)90028-1

[9] AuCoin DP, Colletti KS, Xu Y, Cei SA, Pari GS Kaposi's sarcoma-associated herpesvirus (human herpesvirus 8) contains two functional lytic origins of DNA replication. J Virol. 2002; 76:7890–7896. 12097603 10.1128/JVI.76.15.7890-7896.2002 PMC136389

[10] Rickinson AB, Kieff E (2001) Epstein-Barr Virus In: Knipe DM, Howley PM, Griffin DE, Martin MA, Lamb RA et al, editors. Fields—Virology. Philadelphia: Lippincott Williams & Wilkins pp. 2575–2627.

[11] Schulz TF Kaposi's sarcoma-associated herpesvirus (human herpesvirus-8). J Gen Virol. 1998; 79:1573–1591. 9680119 10.1099/0022-1317-79-7-1573

[12] Grundhoff A, Ganem D Inefficient establishment of KSHV latency suggests an additional role for continued lytic replication in Kaposi sarcoma pathogenesis. J Clin Invest. 2004; 113:124–136. 14702116 10.1172/JCI200417803 PMC300762

[13] Ganem D KSHV and Kaposi's sarcoma: the end of the beginning? Cell. 1997; 91:157–160. 9346233 10.1016/s0092-8674(00)80398-0

[14] Cesarman E, Mesri EA, Gershengorn MC Viral G protein-coupled receptor and Kaposi's sarcoma: a model of paracrine neoplasia? J Exp Med. 2000; 191:417–421. 10662787 10.1084/jem.191.3.417 PMC2195817

[15] Martin DF, Kuppermann BD, Wolitz RA, Palestine AG, Li H, Robinson CA Oral ganciclovir for patients with cytomegalovirus retinitis treated with a ganciclovir implant. Roche Ganciclovir Study Group. N Engl J Med. 1999; 340:1063–1070. 10194235 10.1056/NEJM199904083401402

[16] Virgin HW IV, Latreille P, Wamsley P, Hallsworth K, Weck KE, Dal Canto AJ et al Complete sequence and genomic analysis of murine gammaherpesvirus 68. J Virol. 1997; 71:5894–5904. 9223479 10.1128/jvi.71.8.5894-5904.1997 PMC191845

[17] Barton E, Mandal P, Speck SH Pathogenesis and Host Control of Gammaherpesviruses: Lessons from the Mouse. Annu Rev Immunol. 2011; 29:351–397. 10.1146/annurev-immunol-072710-081639 21219186

[18] Adler H, Steer B, Freimuller K, Haas J Murine gammaherpesvirus 68 contains two functional lytic origins of replication. J Virol. 2007; 81:7300–7305. 17442722 10.1128/JVI.02406-06 PMC1933304

[19] Gong D, Qi J, Arumugaswami V, Sun R, Deng H Identification and functional characterization of the left origin of lytic replication of murine gammaherpesvirus 68. Virology. 2009; 387:285–295. 10.1016/j.virol.2009.02.029 19285330 PMC2715915

[20] Flach B, Steer B, Thakur NN, Haas J, Adler H The M10 locus of murine gammaherpesvirus 68 contributes to both the lytic and the latent phases of infection. J Virol. 2009; 83:8163–8172. 10.1128/JVI.00629-09 19493995 PMC2715785

[21] Liu L, Usherwood EJ, Blackman MA, Woodland DL T-cell vaccination alters the course of murine herpesvirus 68 infection and the establishment of viral latency in mice. J Virol. 1999; 73:9849–9857. 10559297 10.1128/jvi.73.12.9849-9857.1999 PMC113034

[22] Stevenson PG, Belz GT, Castrucci MR, Altman JD, Doherty PC A gamma-herpesvirus sneaks through a CD8^+^ T cell response primed to a lytic-phase epitope. Proc Natl Acad Sci USA. 1999; 96:9281–9286. 10430934 10.1073/pnas.96.16.9281 PMC17771

[23] Tibbetts SA, Loh J, van Berkel V, McClellan JS, Jacoby MA, Kapadia SB et al Establishment and maintenance of gammaherpesvirus latency are independent of infective dose and route of infection. J Virol. 2003; 77:7696–7701. 12805472 10.1128/JVI.77.13.7696-7701.2003 PMC164792

[24] Huang F, Wagner M, Siddiqui MA Structure, expression, and functional characterization of the mouse CLP-1 gene. Gene. 2002; 292:245–259. 12119119 10.1016/s0378-1119(02)00596-6

[25] Ouchida R, Kusuhara M, Shimizu N, Hisada T, Makino Y, Morimoto C et al Suppression of NF-kappaB-dependent gene expression by a hexamethylene bisacetamide-inducible protein HEXIM1 in human vascular smooth muscle cells. Genes Cells. 2003; 8:95–107. 12581153 10.1046/j.1365-2443.2003.00618.x

[26] Xue SA, Griffin BE Complexities associated with expression of Epstein-Barr virus (EBV) lytic origins of DNA replication. Nucleic Acids Res. 2007; 35:3391–3406. 17478522 10.1093/nar/gkm170 PMC1904260

[27] Feederle R, Delecluse HJ Low level of lytic replication in a recombinant Epstein-Barr virus carrying an origin of replication devoid of BZLF1-binding sites. J Virol. 2004; 78:12082–12084. 15479851 10.1128/JVI.78.21.12082-12084.2004 PMC523260

[28] Xu Y, Rodriguez-Huete A, Pari GS Evaluation of the lytic origins of replication of Kaposi's sarcoma-associated virus/human herpesvirus 8 in the context of the viral genome. J Virol. 2006; 80:9905–9909. 16973596 10.1128/JVI.01004-06 PMC1617234

[29] Blomen VA, Majek P, Jae LT, Bigenzahn JW, Nieuwenhuis J, Staring J et al Gene essentiality and synthetic lethality in haploid human cells. Science. 2015; 350:1092–1096. 10.1126/science.aac7557 26472760

[30] Dey A, Chao SH, Lane DP HEXIM1 and the control of transcription elongation: from cancer and inflammation to AIDS and cardiac hypertrophy. Cell Cycle. 2007; 6:1856–1863. 17671421 10.4161/cc.6.15.4556

[31] Ou M, Sandri-Goldin RM Inhibition of cdk9 during herpes simplex virus 1 infection impedes viral transcription. PLoS ONE. 2013; 8:e79007 10.1371/journal.pone.0079007 24205359 PMC3799718

[32] Rennekamp AJ, Lieberman PM Initiation of Epstein-Barr virus lytic replication requires transcription and the formation of a stable RNA-DNA hybrid molecule at OriLyt. J Virol. 2011; 85:2837–2850. 10.1128/JVI.02175-10 21191028 PMC3067963

[33] Wang Y, Li H, Chan MY, Zhu FX, Lukac DM, Yuan Y Kaposi's sarcoma-associated herpesvirus ori-Lyt-dependent DNA replication: cis-acting requirements for replication and ori-Lyt-associated RNA transcription. J Virol. 2004; 78:8615–8629. 15280471 10.1128/JVI.78.16.8615-8629.2004 PMC479094

[34] Wang Y, Tang Q, Maul GG, Yuan Y Kaposi's sarcoma-associated herpesvirus ori-Lyt-dependent DNA replication: dual role of replication and transcription activator. J Virol. 2006; 80:12171–12186. 17020951 10.1128/JVI.00990-06 PMC1676287

[35] Loyola A, Almouzni G Histone chaperones, a supporting role in the limelight. Biochim Biophys Acta. 2004; 1677:3–11. 15020040 10.1016/j.bbaexp.2003.09.012

[36] Terhune SS, Moorman NJ, Cristea IM, Savaryn JP, Cuevas-Bennett C, Rout MP et al Human cytomegalovirus UL29/28 protein interacts with components of the NuRD complex which promote accumulation of immediate-early RNA. PLoS Pathog. 2010; 6:e1000965 10.1371/journal.ppat.1000965 20585571 PMC2891856

[37] Adler H, Messerle M, Wagner M, Koszinowski UH Cloning and mutagenesis of the murine gammaherpesvirus 68 genome as an infectious bacterial artificial chromosome. J Virol. 2000; 74:6964–6974. 10888635 10.1128/jvi.74.15.6964-6974.2000 PMC112213

[38] Spekker K, Leineweber M, Degrandi D, Ince V, Brunder S, Schmidt SK et al Antimicrobial effects of murine mesenchymal stromal cells directed against Toxoplasma gondii and Neospora caninum: role of immunity-related GTPases (IRGs) and guanylate-binding proteins (GBPs). Med Microbiol Immunol. 2013; 202:197–206. 10.1007/s00430-012-0281-y 23269418

[39] Sattler C, Steinsdoerfer M, Offers M, Fischer E, Schierl R, Heseler K et al Inhibition of T-cell proliferation by murine multipotent mesenchymal stromal cells is mediated by CD39 expression and adenosine generation. Cell Transplant. 2011; 20:1221–1230. 10.3727/096368910X546553 21176405

[40] Adler H, Messerle M, Koszinowski UH Virus reconstituted from infectious bacterial artificial chromosome (BAC)-cloned murine gammaherpesvirus 68 acquires wild-type properties in vivo only after excision of BAC vector sequences. J Virol. 2001; 75:5692–5696. 11356978 10.1128/JVI.75.12.5692-5696.2001 PMC114283

