# Supplementary material for: Multiple Lytic Origins of Replication Are Required for Optimal Gammaherpesvirus Fitness In Vitro and In Vivo
Source: PLoS Pathog. 2016 Mar 23;12(3):e1005510. doi: 10.1371/journal.ppat.1005510 (PMC4805163; doi:10.1371/journal.ppat.1005510)
Supplement: S1 Text — Figure A: Schematic diagram showing the structure of the recombinant viruses used in this study. Figure B: Replication of oriLyt mutants and corresponding ectopic revertants in vitro. Figure C: MS/MS spectra for selected peptides. Figure D: Confirmation of results from DNA affinity purification and MassSpec by Western Blot. Figure E: Replication of parental virus and Δright oriLyt mutant in vitro after upregulation of Hexim1. Figure F: Confirmation of overexpression of Rbbp4. Table A: Lytic growth of oriLyt mutants in vitro. Table B: Phenotype of oriLyt mutants in vivo. Table C: Peptides detected from selected proteins by MassSpec analysis. (DOCX) [file ppat.1005510.s001.docx]

**
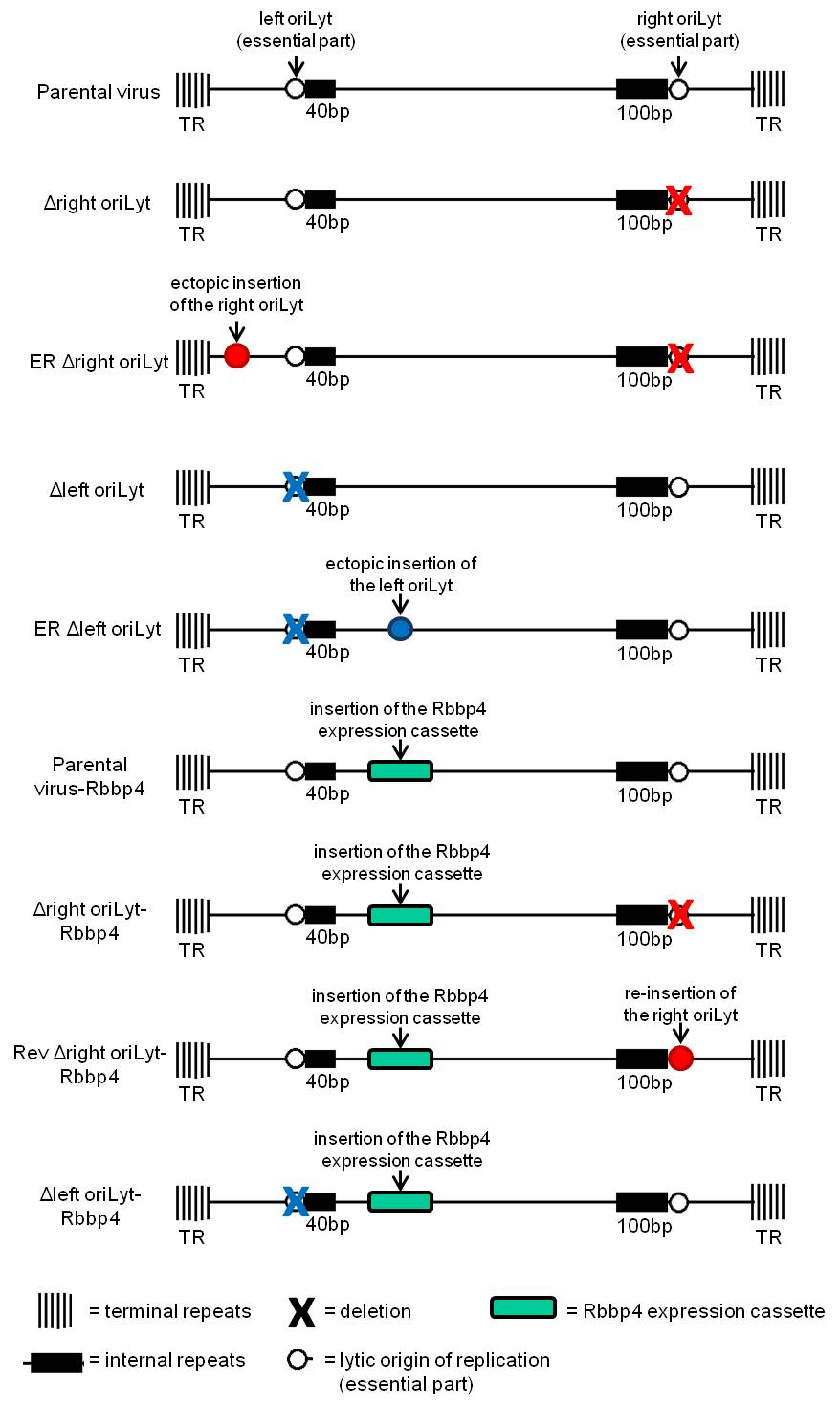
Supplementary Information**

**Figure A:** Schematic diagram showing the structure of the recombinant viruses used in this study. Details on the construction of all recombinant MHV-68 used in this study can be found in the section “Supplementary Methods”.

**A B**

**Figure B:** Replication of oriLyt mutants and corresponding ectopic revertants in vitro. The endothelial cell line MHEC was infected with the indicated viruses at an MOI of 0.01. Cells and cell culture supernatants were harvested at different time points p.i., and titers were determined by plaque assay on BHK-21 cells. Results for the left oriLyt are depicted in (A) and results for the right oriLyt are depicted in (B). Data shown are the means ± SD from three independent experiments.

**A B**

AENLQLLTENELHR GEFGGFGSVSGK

**Figure C:** MS/MS spectra for the peptide that matches Hexim1 (Mascot score: 29) (A) and for one of the four peptides that match Rbbp4 (Mascot score: 56) (B). The peptide sequences are shown at the top of each spectrum.


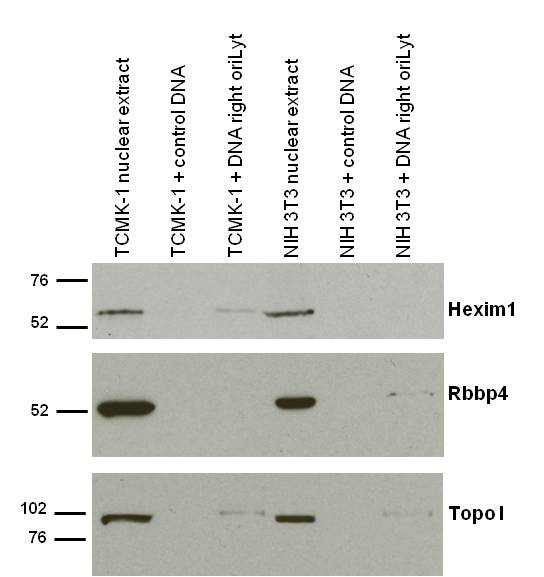


**Figure D:** Confirmation of results from DNA affinity purification and MassSpec. Western Blots for Hexim1 and Rbbp4 with samples from both TCMK-1 and NIH 3T3 cells after DNA affinity purification were performed to confirm binding of Hexim1 or Rbbp4 to DNA of the right oriLyt in the corresponding cell line. Samples purified with DNA of the right oriLyt of MHV-68, whole nuclear extracts before purification and control samples purified with an unspecific DNA sequence are shown. As a control, the samples were also probed with an antibody against Topoisomerase I that had been identified by MassSpec in samples from both cell lines. After DNA affinity purification, Hexim1 was only detected in samples from TCMK-1 cells purified with the specific oriLyt DNA but not in NIH 3T3 cells whereas Rbbp4 was only found in DNA affinity purified samples from NIH 3T3 cells. Topoisomerase I could be detected in samples from both cell lines. No bands were found in any sample purified with unspecific control DNA.

**A**   **B**

**Figure E:** Replication of parental virus and Δright oriLyt mutant in vitro after upregulation of Hexim1: To upregulate Hexim1, NIH 3T3 cells were plated and after 4 hours stimulated with 10 mM HMBA or left untreated. 24 hours later, cells were infected with the indicated viruses at an MOI of 0.01 for 1 hour. After removing the inoculum, cells were incubated with fresh medium without HMBA (A) or with 10 mM HMBA (B) at 37°C and 5% CO_2_ until the supernatants together with the cells were harvested at different time points after infection. Virus titers were determined by plaque assay. Data shown are means + SD of three independent experiments.


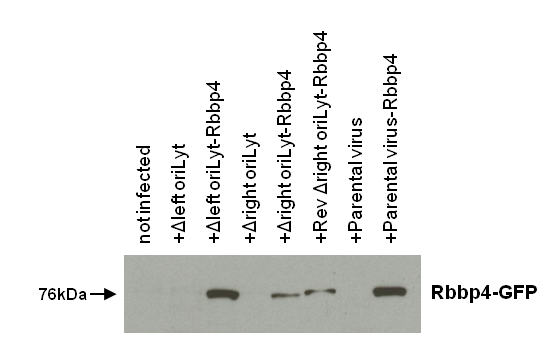


**Figure F:** Confirmation of overexpression of Rbbp4. Overexpression of Rbbp4 was accomplished by constructing recombinant MHV-68 expressing the cellular protein Rbbp4 as a GFP-fusion protein. TCMK-1 cells were infected with the indicated viruses, and the expression of Rbbp4-GFP was analyzed by Western Blot 48 hours after infection.

| **name** | **cell type** | **tissue of origin** | **mouse strain** | **virus titers Δleft oriLyt** | **virus titers Δright oriLyt** |
| --- | --- | --- | --- | --- | --- |
| MH-S | alveolar macrophage | lung | Balb/c | **=** | **↓** |
| MHEC | endothelial cell | heart | NMRI | **↓** | **↓** |
| SVEC4-10 | endothelial cell | axillary lymphnode | C3H/HeJ | **↓** | **↓** |
| C127 | epithelial cell | mammary gland | RIII | **↓** | **↓** |
| LA-4 | epithelial cell | lung | A/He | **↓** | **↓** |
| MLE12 | epithelial cell | lung | FVB/N | **=** | **=** |
| TCMK-1 | epithelial cell | kidney | C3H/Mai | **↓** | **=** |
| MEF | fibroblast | embryonal | C57BL/6 | **↓** | **↓** |
| NIH 3T3 | fibroblast | embryonal | NIH/Swiss | **=** | **↓** |
| CS16 | stromal cell | bone marrow | C57BL/6 | **=** | **↓** |
| CS22 | stromal cell | bone marrow | Balb/c | **=** | **=** |
| OP9 | stromal cell | embryonal | (C57BL/6 x C3H)F2 –op/op | **=** | **↓** |
| MOVAS | smooth muscle cell | aorta | C57BL/6 | **=** | **=** |

**Table A:** Lytic growth of oriLyt mutants in vitro. The table shows features of the tested cell lines including cell type, tissue of origin and genetic background. It summarizes the results obtained for lytic growth of the virus mutants in various cell lines compared to parental virus. “**↓**” indicates reduced growth; “=” indicates no difference to parental virus.

|  | **Δleft oriLyt** | | **Δright oriLyt** | |
| --- | --- | --- | --- | --- |
|  | **intranasal inoculation** | **intraperitoneal inoculation** | **intranasal inoculation** | **intraperitoneal inoculation** |
| Lytic phase | ↓ | n.d. | ↓↓ | n.d. |
| Latency establishment (viral load) | ↓ | ↓ | ↓ | ↓ |
| Reactivation (in general) | ↓ | ↓ | ↓↓ | ↓ |
| Reactivation efficiency (in relation to viral load) | = | = | ↓ | = |

**Table B:** Phenotype of oriLyt mutants in vivo. The table shows an overview of lytic replication, latent viral load and reactivation after intranasal or intraperitoneal inoculation, compared to parental virus. “**↓**” indicates reduced growth; “=” indicates no difference to parental virus; n.d., not done.

| **Selected proteins found specifically only in one cell line** | | |
| --- | --- | --- |
| **Name** | **Peptides identified in NIH 3T3 cells** | **Peptides identified in TCMK-1 cells** |
| Hexim1 | none | R.AENLQLLTENELHR.Q |
| Rbbp4 | R.VINEEYK.I  K.TVALWDLR.N  K.GEFGGFGSVSGK.I  R.RLNVWDLSK.I | none |
|  |  |  |
|  |  |  |
| **Selected proteins found in both cell lines** | | |
| **Name** | **Peptides identified in NIH 3T3 cells** | **Peptides identified in TCMK-1 cells** |
| DNA Topoisomerase I | K.AEEVATFFAK.M  K.LEVQATDREENK.Q  R.TYNASITLQQQLK.E | K.IEPPGLFR.G  R.AVALYFIDK.L  K.AEEVATFFAK.M  K.HLQDLMEGLTAK.V  K.LEVQATDREENK.Q |
| Ku86 | K.FLAPKDK.A  K.IPNPEFQR.L  R.FNSFLEALR.E  K.TLFPLTEVIK.K  K.VGSVNPVENFR.F  R.SNPQVGVAFPYIK.D  K.NQVTAQDVFQDNHEEGPAAK.K | K.IPNPEFQR.L  R.FNSFLEALR.E  K.VGSVNPVENFR.F  R.GDLDSGLDHLKPSFPQK.G  R.QVFSESKDEIALVLYGTDGTDNALAGK.D |
| PARP1 | R.YKPFR.Q  R.EISYLK.K  R.VGTVIGSNK.L  K.AEPGEVVAPK.G  K.GIYFADMVSK.S  K.TLGDFLAEYAK.S  K.AQNELIWNIK.D  K.ALVEYEIDLQK.M  R.KGDEVDGTDEVAK.K  R.TTNFAGILSQGLR.I  R.FYTLIPHDFGMK.K  K.KPPLLNNADSVQAK.V  R.IAPPEAPVTGYMFGK.G  K.MVDPEKPQLGMIDR.W  R.DELGFRPEYSASQLK.G  K.LEQMPSKEDAVEHFMK.L  K.GGAAVDPDSGLEHSAHVLEK.G | R.YKPFR.Q  K.GIYFADMVSK.S  K.TLGDFLAEYAK.S  K.AQNELIWNIK.D  K.ALVEYEIDLQK.M  K.LQLLEDDKESR.Y  K.VFSATLGLVDIVK.G  R.FYTLIPHDFGMK.K  R.QPDVEVDGFSELR.W  K.KPPLLNNADSVQAK.V  K.VVDRDSEEAEVIR.K  R.IAPPEAPVTGYMFGK.G  K.MVDPEKPQLGMIDR.W  R.GGSDDSSKDPIDVNYEK.L  K.GGAAVDPDSGLEHSAHVLEK.G  K.ELLIFNQQQVPSGESAILDR.V |
| hnRNP A3 | R.DYFEK.Y  K.KIFVGGIK.E  K.EDTEEYNLR.D  K.IETIEVMEDR.Q  K.WGTLTDCVVMR.D  R.EDSVKPGAHLTVK.K  K.YHTINGHNCEVK.K  K.RGFAFVTFDDHDTVDK.I  K.IFVGGIKEDTEEYNLR.D  R.SSGSPYGGGYGSGGGSGGYGSR.R  K.LFIGGLSFETTDDSLREHFEK.W | K.KIFVGGIK.E  K.EDTEEYNLR.D  K.IETIEVMEDR.Q  R.EDSVKPGAHLTVK.K  K.YHTINGHNCEVK.K  K.YGKIETIEVMEDR.Q  K.IFVGGIKEDTEEYNLR.D  R.SSGSPYGGGYGSGGGSGGYGSR.R  R.GFAFVTFDDHDTVDKIVVQK.Y  K.LFIGGLSFETTDDSLREHFEK.W |

**Table C:** Peptides detected from selected proteins by MassSpec analysis.

**Supplementary Methods:**

*Cell lines*

BHK-21 cells (ATCC: CCL-10) were grown in Glasgow-MEM (PAN Biotech, Aidenbach, Germany) supplemented with 5% fetal calf serum (FCS; PAN Biotech, Aidenbach, Germany), 5% tryptose phosphate broth, 2 mM L-glutamine, 100 U/ml Penicillin and 100 μg/ml Streptomycin. REF-Cre cells [1] were maintained in DMEM High Glucose (Gibco, Darmstadt, Germany) supplemented with 10% FCS, 2 mM L-Glutamine, 100 U/ml Penicillin, 100 μg/ml Streptomycin and G418. NIH 3T3 cells (ATCC: CRL-1658) were grown in DMEM High Glucose (Gibco, Darmstadt, Germany) supplemented with 10% FCS, 2 mM L-Glutamine, 100 U/ml Penicillin and 100 μg/ml Streptomycin (=NIH 3T3 medium). Additionally, the following cell lines were used in this study: MH-S (ATCC: CRL-2019), MHEC (DSMZ: ACC 336), SVEC4-10 (ATCC: CRL-2181), C127 (ATCC: CRL-1616), MLE12 (ATCC: CRL-2110), TCMK-1 (ATCC: CCL-139), MEF (ATCC: SCRC-1008), OP9 (ATCC: CRL-2749), MOVAS (ATCC: CRL-2797), LA-4 (ATCC: CCL-196), mMSC CS16 [2], and mMSC CS22 [3]. MH-S cells were grown in RPMI (Gibco, Darmstadt, Germany) supplemented with 10% FCS, 2mM L-Glutamine, 100U/ml Penicillin, 100µg/ml Streptomycin, and 50µM 2-Mercaptoethanol (Bioconcept, Allschwil, Switzerland). All other cell lines were grown in NIH 3T3 medium. For cultivation of MOVAS cells, 0,2 mg/ml G418 were added to the culture medium. For cultivation of OP9, mMSC CS16 and mMSC CS22, the medium was supplemented with additional 10% FCS to make a final concentration of 20% FCS.

*Protein identification by nanoHPLC-ESI-MS/MS*

Protein identification was performed by Proteome Factory (Proteome Factory AG, Berlin, Germany). The LCMS system consisted of an Agilent 1100 nanoHPLC system (Agilent, Waldbronn, Germany), PicoTip electrospray emitter (New Objective, Woburn, MA) and a QExactive mass spectrometer (ThermoFisher Scientific, Bremen, Germany). Protein spots were in-gel digested by trypsin (Promega, Mannheim, Germany) and analysed by nanoHPLC-ESI-MS/MS. Peptides were first trapped and desalted on the enrichment column (Zorbax 300SB-C18, 0.3 mm x 5 mm, Agilent) for five minutes (solvent: 2.5% acetonitrile/0.5% formic acid), then separated on a Zorbax 300SB-C18, 75 µm x 150 mm column (Agilent) using a linear gradient from 10% to 32% (solvent A: 5% acetonitrile in water, solvent B: acetonitrile, both with 0.1% formic acid). Ions of interest were data-dependently subjected to MS/MS according to the expected charge state distribution of peptide ions. Proteins were identified by database search against the NCBInr protein database (National Center for Biotechnology Information, Bethesda, USA) limited to murine proteins (Mus musculus) using MS/MS ion search of the Mascot search engine (Matrix Science, London, England). The search parameters included + 5 ppm precursor mass tolerance and + 0.02 Da fragment mass tolerance and allowed one missed cleavage and the following variable modifications: oxidation (M), propionamide (C) and carbamidomethyl (C). Static modifications (e.g. Cys alkylation) were not considered. A significance threshold of p < 0.05 (mascot default) was used. The corresponding Mascot score was 27.

*Chromatin ImmunoPrecipitation (ChIP)*

For ChIP analysis 5 x 10^6^ TCMK-1 cells (for Hexim1 ChIP) or 5 x 10^6^ NIH 3T3 cells (for Rbbp4 ChIP) were infected with MHV-68 parental virus at an MOI of 0.1. After 72 hours incubation at 37°C the cells were washed in ice cold PBS and treated with 1% formaldehyde for 10 min at room temperature (RT). Glycine was added to a final concentration of 125 mM for 5 min at RT. Cells were washed twice in ice cold PBS, 5 ml ice cold PBS were added and the cells were detached from the flask using a cell scraper. Cells were pelleted and resuspended in RIPA buffer containing protease inhibitors (Complete Mini Protease Inhibitor Tablet, Roche, Mannheim, Germany). Chromatin was fragmented by sonication using a Bioruptor (Diagenode, Liège, Belgium). After centrifugation at 12.500x g for 10 min at 4°C aliquots of the supernatant were stored at -80°C. For immunoprecipitation 40µl of the chromatin solution were adjusted to a volume of 500 µl with dilution buffer (16.7 mM Tris-HCI, pH 8.1, 1.2 mM EDTA, 1.1 % Triton X-100, 167 mM NaCl, 0.01 % SDS, Protease Inhibitor) containing salmon sperm DNA and BSA. The input samples were precleared by incubation with ProteinG MicroBeads (Miltenyi Biotech, Bergisch-Gladbach, Germany) and passing the lysate over a µ Column placed in a µMACS separator. The flowthrough was collected as pre-cleared sample. The pre-cleared input samples were incubated with 1µg of a rabbit anti-Hexim1 antibody (Abcam, Cambridge, UK) or 1µg of a rabbit anti-Rbbp4 antibody (Novus Biologicals, Cambridge, UK), respectively, for 90 min at 4°C. The corresponding isotype matched antibody was used as a control. Magnetic ProteinG MicroBeads pre-incubated with salmon sperm DNA and BSA were added for 1 hour at 4°. Immunoprecipitates were mixed with blocking buffer (PBS, 1% skim milk) and administered to magnetic purification using µ Columns and a µMACS separator. Columns were washed three times with low salt wash buffer (20 mM Tris-HCI, pH 8.1, 2 mM EDTA, 1% Triton X-100, 150 mM NaCl, 0.1% SDS), twice with high salt wash buffer (20 mM Tris-HCI, pH 8.1, 2 mM EDTA, 1% Triton X-100, 500 mM NaCl, 0.1% SDS), twice with LiCl wash buffer (10 mM Tris-HCI, pH 8.1, 1 mM EDTA, 250 mM LiCl, 1% NP-40, 1% sodium deoxycholate) and twice with TE buffer. 50µl hot elution buffer (100 mM NaHCO3, 1% SDS) were added to the column to elute DNA and flowthrough was collected. After 1 min incubation time another 50µl elution buffer were added and flowthrough was pooled with the first eluate. This step was repeated another two times to reach a final volume of 200µl eluate. As a control 50µl of pre-cleared input sample were mixed with 150µl hot elution buffer and then processed like the ChIP eluates. The samples were treated with 200mM NaCl (final concentration) to reverse the crosslink followed by a protein degradation step with Proteinase K for 2 hours at 65°C. The DNA was purified using the QIAmp DNA Mini Kit (Qiagen, Hilden, Germany). Input and ChIP DNA were analyzed by quantitative real-time PCR using the ABI 7300 Real Time PCR System (Applied Biosystems, Foster City, CA) with a primer pair amplifying a 151 bp sequence in the DNA of the right oriLyt and a primer pair amplifying a 198 bp sequence in the DNA of the left oriLyt. A third primer pair amplifying a 194 bp sequence within the ORF23 coding region was used as a control.

*Plasmid construction*

For this study, we constructed eight different recombinant MHV-68:

1) The mutant virus Δleft oriLyt lacking the essential part of the left oriLyt was created by ET-cloning [1,4]. For this purpose, the essential part of the left oriLyt of MHV-68 was first replaced with a tetracycline (Tet) resistance gene flanked by FRT sites. Subsequently, the Tet resistance cassette was removed by FLP-mediated recombination, resulting in a deletion between nucleotides 26232 and 26373 which disrupts the oriLyt. The procedure left a small residual insert consisting of an FRT site and short vector sequences in the disrupted region.

2) We constructed a recombinant virus with an ectopic insertion of the left oriLyt (ER Δleft oriLyt). Since the left oriLyt is located inside the M5 region of MHV-68, we wanted to differentiate between effects caused by disruption of the left oriLyt or by disruption of the putative M5 ORF. Therefore, we introduced a point mutation in the ectopically inserted M5 region so that the oriLyt is restored but M5 is disrupted by a stop codon. For that purpose, a 1.1kb fragment of MHV-68 (nucleotide positions 25695 to 26803) containing the left oriLyt and the M5 region with a stop codon, was generated by a site-directed mutagenesis with two subsequent PCRs using a PCR-mediated overlap extension with mutagenic primers according to the method described by Heckman et al. [5]. The PCR product was cloned into the PmlI site (nucleotide position 46347 of the MHV-68 genome) of the plasmid pST76K-SR containing a 4.0 kb SphI-SacI fragment of MHV-68 (nucleotides 44301 to 48346). As a result, the 1.1kb fragment is flanked on both sides by homologous sequences as needed for homologous recombination during the two-step mutagenesis procedure [1,6].

3) The construction of a mutant (Δright oriLyt) with a deletion of the essential part of the right oriLyt lacking nucleotide positions 101530 to 101731, has been described previously [7].

4) We constructed a recombinant virus with an insertion of the oriLyt which restores the right oriLyt at an ectopic position (ER Δright oriLyt). For that purpose, a 863 bp SmaI - EagI fragment of MHV-68 (nucleotide positions 101139 to 102002) was cloned blunt end into the BglII site (nucleotide position 3846 of the MHV-68 genome) of the plasmid pST76K-SR M1/M2 [8]. As a result, the 863 bp fragment is flanked on both sides by homologous sequences (position 2406-3846 as 5’ flank and position 3847-6261 as 3’ flank) as needed for homologous recombination during the two-step mutagenesis procedure.

5) For overexpression of the cellular protein Rbbp4, recombinant MHV-68 expressing Rbbp4 from an intergenic expression cassette (Parental virus-Rbbp4; Δleft oriLyt-Rbbp4; Δright oriLyt-Rbbp4) were constructed by the two-step mutagenesis procedure. To this end, an expression cassette containing the coding sequence of Rbbp4, C-terminally GFP-tagged and driven by a CMV promoter, was excised from an Rbbp4-expression plasmid (OriGene Technologies, Rockville, MD, USA) and inserted into the PmlI site (nucleotide position 46347 of the MHV-68 genome) of the plasmid pST76K-SR already containing a 4.0 kb SphI-SacI fragment of MHV-68 (nucleotide positions 44301 to 48346). As a result, the Rbbp4 expression cassette is flanked on both sides by homologous sequences as needed for homologous recombination during the two-step mutagenesis procedure.

6) A revertant of the Δright oriLyt-Rbbp4 recombinant virus was constructed by the two-step mutagenesis procedure [1,6], using an unmutated 8.4 kb HindIII fragment of MHV-68 (nucleotides 95677 to 104034), resulting in re-insertion of the right oriLyt (Rev Δright oriLyt-Rbbp4).

**Supplementary References:**

1. Adler H, Messerle M, Wagner M, Koszinowski UH Cloning and mutagenesis of the murine gammaherpesvirus 68 genome as an infectious bacterial artificial chromosome. J Virol. 2000; 74:6964-6974.

2. Spekker K, Leineweber M, Degrandi D, Ince V, Brunder S, Schmidt SK et al. Antimicrobial effects of murine mesenchymal stromal cells directed against Toxoplasma gondii and Neospora caninum: role of immunity-related GTPases (IRGs) and guanylate-binding proteins (GBPs). Med Microbiol Immunol. 2013; 202:197-206.

3. Sattler C, Steinsdoerfer M, Offers M, Fischer E, Schierl R, Heseler K et al. Inhibition of T-cell proliferation by murine multipotent mesenchymal stromal cells is mediated by CD39 expression and adenosine generation. Cell Transplant. 2011; 20:1221-1230.

4. Zhang Y, Muyrers JP, Testa G, Stewart AF DNA cloning by homologous recombination in Escherichia coli. Nat Biotechnol. 2000; 18:1314-1317.

5. Heckman KL, Pease LR Gene splicing and mutagenesis by PCR-driven overlap extension. Nat Protoc. 2007; 2:924-932.

6. Messerle M, Crnkovic I, Hammerschmidt W, Ziegler H, Koszinowski UH Cloning and mutagenesis of a herpesvirus genome as an infectious bacterial artificial chromosome. Proc Natl Acad Sci USA. 1997; 94:14759-14763.

7. Adler H, Steer B, Freimuller K, Haas J Murine gammaherpesvirus 68 contains two functional lytic origins of replication. J Virol. 2007; 81:7300-7305.

8. El-Gogo S, Staib C, Lasarte JJ, Sutter G, Adler H Recombinant murine gammaherpesvirus 68 (MHV-68) as challenge virus to test efficacy of vaccination against chronic virus infections in the mouse model. Vaccine. 2007; 25:3934-3945.
